# Supplementary material for: Starting the SToP trial: Lessons from a collaborative recruitment approach
Source: PLoS One. 2022 Nov 17;17(11):e0273631. doi: 10.1371/journal.pone.0273631 (PMC9671300; doi:10.1371/journal.pone.0273631)
Supplement: S1 File — (DOCX) [file pone.0273631.s001.docx]

# **Appendix A**

## Yarning Session Questions for Community Members

On behalf of Telethon Kids Institute, thank you for taking the time to yarn with me today to discuss the SToP trial recruitment process. This yarn should take appropriately 30 minutes and if at anytime you would like to stop the yarn, please let me know. We are keen to hear how you found the recruitment process and if there is any way we can make it better. We are also interested in how useful you found the information from the flipchart. You do not have to answer any questions that you are not comfortable with. With your consent this interview will be audio-recorded and all information will be de-identified and remain confidential.

**Four or Five Broad Themes: Example questions only, Aboriginal person to guide yarning session**

1. **Leading question into the yarning session?**
   - This question will be guided by an Aboriginal person and could be related to family/community life, skin infections or health in general.
2. **Cultural appropriateness of recruiting process**
   - Can you please tell me what you remember when recruiting staff visited your community/house to yarn about the SToP trial and skin infections?
   - What did you find good or not good about when you spoke to recruiting staff?
   - Did having an Aboriginal person explaining the information about the study help you to decide to participate?
3. **Flip Chart**
   - Did you think the flipchart that recruiting staff showed you was interesting and culturally appropriate?
   - Did you find anything out about skin infections that you didn’t know before? Did the flip-chart information help to decide to participate in the study?
   - What do you think could be done better or differently about the flipchart next time to explain why the trial is important?
4. **What can be done differently or better next time?**
   - Are there other suggestions do you have on what could be done differently or better next time when speaking with families and recruiting for research projects?
   - What other comments would you like to share about your experience when recruiting staff visited your house to yarn about the SToP trial and skin infections?

Thank you again for your time, I appreciate you talking to me today. Your personal information will be kept confidential, your comments may be used in publications and presentations.

**Recruitment Evaluation Semi-structured interview questions for the partner organisation**

On behalf of Telethon Kids Institute, thank you for taking the time to meet with me today to discuss the SToP trial recruitment process. This interview should take appropriately 30 minutes and if at anytime you would like to stop the interview, please let me know. You do not have to answer any questions that you are not comfortable with. With your consent this interview will be audio-recorded and all information will be de-identified and you will remain anonymous.

1. Please describe how and when you were involved in the recruitment process for the SToP trial?
2. How long did you attend the one-day educational workshop for?

If no attendance, why did you not attend?

1. What did you find effective about this workshop for learning about skin infections?
2. What suggestions would you have for improvement?
3. How many communities did you visit? What were some of the differences between communities you visited in terms of how the community members engaged with you?
4. Was the flipchart used in the recruitment process effective for informing community members about the SToP trial and skin infections? Why/Why not?
5. What other strategies or educational resources would work well to inform community members?
6. What do you think worked well with the recruitment process?
7. What were the challenges you encountered in the recruitment process when visiting communities or challenges in general?
8. What strategies do you think might help overcome these challenges for future research projects?
9. How has working in partnership with Telethon influenced participation into the SToP trial?
10. For those community members who declined to be involved, what were some of the reasons for saying No?
11. Other research suggests that when some community members decline to be involved, this can influence other community members’ decision to be involved. Do you think this happened when you were recruiting participants? Why/Why not?
12. What would you do differently about this process next time if you were to be involved in recruiting participants into a study like the SToP trial?
13. What further comments you would like to share about your experience of the recruitment process?

Thank you again for your time, I appreciate you talking to me today. Your personal information will be kept private however your comments may be used in publications and presentations, but you will not be identified

**Recruitment Evaluation Semi-structured interview questions for Telethon Kids Institute staff**

On behalf of Telethon Kids Institute, thank you for taking the time to meet with me today to discuss the recruitment process. This interview should take appropriately 30 minutes and if at anytime you would like to stop the interview, please let me know. You do not have to answer any questions that you are not comfortable with. With your consent this interview will be audio-recorded and all information will be de-identified and you will remain anonymous.

1. Please describe how and when you were involved in the recruitment process for the SToP trial?
2. How long did you attend the one-day educational workshop for?

If no attendance, why did you not attend?

1. How effective was the training for learning about skin infections? Please explain your answer.
2. What do you think worked well with the recruitment process?
3. What were the challenges you encountered during your involvement with the recruitment process?
4. What strategies do you think might help overcome these challenges for future research projects?
5. In what way has working in partnership with Nirrumbuk influenced participation into the SToP trial?
6. What would you do differently about recruitment next time if you were to be involved in recruiting for research?
7. Do you have any further comments you would like to share about your experience of the recruitment process?

Thank you again for your time, I appreciate you talking to me today. Your personal information will be kept private however your comments may be used in publications and presentations, but you will not be identified.
