# Supplementary material for: Starting the SToP trial: Lessons from a collaborative recruitment approach
Source: PLoS One. 2022 Nov 17;17(11):e0273631. doi: 10.1371/journal.pone.0273631 (PMC9671300; doi:10.1371/journal.pone.0273631)
Supplement: S2 File — (DOCX) [file pone.0273631.s002.docx]

# **Appendix B – Outlining how themes emerged from the data**

| **Codes** | **Emerging Themes** | **Number of Quotes Relating to Themes** | **Themes presented as quotes in Manuscript** |
| --- | --- | --- | --- |
| 1. **Barriers** |  |  |  |
|  | Community and House layouts | 4 |  |
|  | Cultural issues | 5 |  |
|  | Declined and influencing others | 1 |  |
|  | Ethics | 2 |  |
|  | Family Structure | 2 |  |
|  | Funding | 4 |  |
|  | Issue with word Scabies | 4 |  |
|  | **Judgment and stigma** | **10** | **Issues were discussed around the stigma of skin infections in community and the shame involved** |
|  | Families not in Community | 3 |  |
|  | **Paperwork/Operational Logistics** | **9** | **Organisational level challenges relating to miscommunication and paperwork logistics was a strong theme emerging from staff members** |
|  | School attendance | 2 |  |
|  | **Timeframes** | **12** | **The protracted process of 12 months to conduct recruitment in a remote context.** |
|  | Telethon Not Present during Recruitment | 3 |  |
|  | Weather | 2 |  |
| 1. **Facilitators** |  |  |  |
|  | Community Connection | 11 | **When individuals conducting recruitment had connections with community members, it was easier to build relationships and trust which resulted in a quicker and easier process** |
|  | Local Community Knowledge | 6 |  |
|  | Collaborative Partnerships | 23 | **The partnership between Telethon Kids Institute and local organisation conducting recruitment emerged as the critical success factor where participants believed this was a culturally appropriate and respectful way to conduct research in remote communities. This partnership had resulted in high participation rates** |
| 1. **Flipchart** |  | 12 | **The flipchart was considered a culturally appropriate intervention and visual resource for explaining to the families about skin infections** |
| 1. **Future Strategies** |  |  |  |
|  | Branding & Communication | 18 | **Team Branding on T Shirts and Posters was considered an appropriate way for researchers to communicate with communities. This strategy allows community members to know who is coming into community and what research project they are working on** |
|  | Correct skills for research | 4 |  |
|  | Group Sessions | 2 |  |
|  | Language | 2 |  |
|  | Local Community Members | 16 | **Utilising and employing local community members for recruiting into research was a major strategy emerging from the data.** |
|  | Timeframes | 4 |  |
