# Supplementary material for: Starting the SToP trial: Lessons from a collaborative recruitment approach
Source: PLoS One. 2022 Nov 17;17(11):e0273631. doi: 10.1371/journal.pone.0273631 (PMC9671300; doi:10.1371/journal.pone.0273631)
Supplement: S3 File — (DOCX) [file pone.0273631.s003.docx]

# S3. File – Minimum Data Set

## Participant 1

Interviewer: So, on behalf of Telethon Kids, um, thank you for speaking with me today, about my evaluation project, and to just discuss the recruitment process for the SToP Trial. Um, this interview should take about thirty minutes, give, or take, and if any time you would like to stop, um, you are free to stop, or you don’t want to answer any questions, that’s fine. Um, you have consented to the interview in being audio recorded, so the audio recorder has been switched on

Perfect, yup.

Interviewer: So, when you were working with the um, the Local Organisation staff, did you, were you involved in any actual consenting families?

Yes, there was a couple. Um, so [pause] I think in both communities, there was a couple of cases where, um, the Local Organisation staff or the clinic staff had identified family’s um that could join were doing the surveillance, and the Local Organisation staff was there as well, and so we um got consent from the parents and did surveillance on the child immediately

Interviewer: And at that time, did you use the flip chart?

No

Interviewer: And was it because it wasn’t available, or…?

It was available, I kind of didn’t really feel like we needed it. I think the verbal explanations we used seemed sufficient. The parent understood and we also, yeah, we were trying to be kind of quick to be honest because there was more children in the room that we had to consent and quickly do the surveillance

Interviewer: And did the Local Organisation staff, did they guide the conversation with the parents?

In those situations, I would say no the parent was kind of asking me questions directly, and so I responded how I thought would be appropriate and just ask them if that was okay and did, they understand that the Local Organisation, like, EHW1 was there as well

Interviewer: Awesome, thank you. Um, In August 2018, there was a one-day—an educational workshop held with the Local Organisation staff to explain healthy skin and to talk about the flip chart, the flip chart was used which was the resource that was used by them

No, I didn’t.

Interviewer: Um so what do you think worked well with the recruitment process from your involvement and your experience?

Um, I think it was really good, um. [sighs] So I-I guess EHW1 was a key person. I think she really um drove the whole process from the Local Organisation side and just the local knowledge that she had and the relationships that she had in community was really the only way that we could identify families that weren’t in the trial, yes so that was really good. That's kind of [Community Name] and then in [Community Name], I think again just having like [EHW names], I think it was were there from Local Organisation, and the first day they really weren’t sure how they were going to find the families we had sort of flagged that we hadn’t consented but they just went out into the community and spoke to people, sort of worked out who those people were and how they could approach them and then on the second day they managed to get I can’t remember how many it was somewhere between ten and twenty new consents so they did really well on the second day after they got that information, you know the local information about how to approach those people.

Interviewer: Do you think that having the school to your knowledge, has the school been kind of driving any of the recruitment or giving suggestions or to people who might?

Yeah, and the school obviously helped us identify kids that we knew who the kids were in the class that weren’t on our list, and they helped us to you know, get their names, and the Local Organisation staff approached families.

Interviewer: And what have been the challenges that you’ve particularly encountered with your involvement or that you think have been kind of barriers for the recruitment process?

This is probably on the ground so [Community Name] worked very well we really knew who the—the student attendance was really good we knew that we had consent of most of the students before we started, so there was only a handful and as I said, the Local Organisation staff kind of went out and got the local knowledge about that on the first day and then the second day they approached the families, so that was really good, and we got like, eighty, ninety percent involvement in that community. In Community Name it was uh really a lot more difficult, and probably the main barriers were um just it was a bigger community, there was more um fluctuation in attendance in the kids that were present in community, the time that kids were at school, um and the school was less involved in helping us identify who those kids that were missing were, they really didn’t know, they had a lot more kids on their enrolment list I think that sometimes attendance at school for a short time and then they would go away for months so we really didn’t have a strong idea of who the kids were that we needed and whether they were in community so that was really difficult and it seemed like they didn’t attend the school, um for the whole week they maybe had two days a week where they would attend so it was getting that local knowledge about what days kids were at school, um that I think would help next time we know you have to kind of focus on the first part of the week, and the kids also went home at lunchtime, um and some didn’t return, and particularly in the senior school, in Community Name, a lot of the kids were involved in other programs, like TAFE um and other school-based programs where they weren’t at school for a number of days of the week, so some were on a fishing excursion ...

Interviewer: And do you think a challenge is also, um [pause] it could be the trends of the families in community?

Yeah, so um, we know that at the time we were in Community Name, a lot were in [community name], a lot were in Community Name, some were in [Name of Town], um, there had been—there was sorry business going on in [community name] and um related to a family in [community name], um so there was a lot of yeah, entire family’s um that weren’t present. There was also conjunctivitis outbreak in the school, so a lot of the kids were kept home

Interviewer: No, you’ve—you’ve an—

[laughs] I’m trying to remember, I think—

Interviewer: You’ve—you’ve answered it, so the—

Interviewer: And you alluded to this when you answered the question about what strategies do you think might overcome some of the challenges for re—for future research projects in this context of trying to recruit?

What strategies [pause, then sighs]

Interviewer: So, you mentioned before having the local knowledge—

Yeah, so—

Interviewer: Like beforehand

Yeah, I think—I mean obviously being accompanied by a local person on the ground [laughs] that can go out into community and help you identify who the families are that are missing or who might want to be involved that hasn’t heard about the research (Interviewer: yeah), um that’s kind of essential (Interviewer: yeah) obviously.

Interviewer: And when you say local person on the ground, would, could that be a, uh, community member, not just, like a—

Yeah, definitely, so when we were there as well, there was a couple of students um that were on a TAFE program um at the clinic for the Aboriginal health worker program and we spoke to them, thinking maybe next visit that they might help us do some of the SToP Trial work and they would be really key to knowing which kids weren’t there and identifying the families and that kind of thing. Um, so somebody like that I think would really help.

Interviewer: And for like future research projects, do you think having knowledge of what’s going on in community, um would that be a good strate—like, to always, um—what—know what’s going on, do you think that would be a good strategy um.

Yeah, I mean uh, definitely. Um, I guess ‘cause when we had those fifty kids that we had consented that we couldn’t find as well, but you know, the ones that we knew sh—thought should have been at school and they weren’t there , and a lot of that was because of these events, so we knew the family that was associated with the sorry business and we knew the ones that had other end-of-the-week you know travel to another community, we knew the ones that had conjunctivitis that kind of thing...

Interviewer: And, in what way in has working in partnership with Local Organisation influenced participation into the SToP trial, do you think?

I think it’s essential, and I think we would have got proper, I just think we wouldn’t have gotten the consents without Local Organisation. We didn’t have the capacity to go out and do it ourselves, and of course that’s not appropriate for us to go out into the community and having it done before we got there, it was key, I think.

Interviewer: What would you do differently about recruitment next time if you were to be involved in recruiting for research?

Um, I haven’t told you as well about the flip charts, ‘because I originally did the first iteration of the flip chart and then [TKI staff member] had a go at it and kind of finished it off and we did spend quite a lot of time doing that and looking back now, you know, I don’t think it was really used. We did use it when we were talking to the students at the assemblies and that kind of thing, so it’s probably useful when you’re talking to a group of people. I don’t know if Local Organisation used it um in their initial visits if they were talking to groups of people, but I don’t think [EHW names] used it when they went out to people unless they had a smaller version, I didn’t’ see them carrying it but I think just more consultation with someone from community about the best way to inform and engage with community members about the trial before you get there so they have that understanding...

## Participant 2

Interviewer: Okay, so I've turned on the audio recorder, and on behalf of Telethon Kids Institute, thank you for taking the time to meet with me today, to discuss the um, the SToP Trial recruitment process, so we can understand what the barriers and the facilitators were to recruiting um and also to see if the flip chart was um effective. It should only take about thirty minutes, and if you want to stop the interview, feel free, if you want to ask any questions, or if you want--don't want to answer any questions, please feel free. Um, and with your consent, this interview is being audio recorded.

Interviewer: Were you involved in the early meetings around the partnership deciding (P2: Yes) Local Organisation to be involved?

So we had some partnership steering group meetings where we were discussing the best strategies for recruitment and definitely the directive from our Kimberley partners WACHs and KAMS and Local Organisation was to have it as an Aboriginal-led process, um and them really feeling the need that was going to be the most effective way um and the best way to do this ethically to ensure that community members would be able to provide informed consent and have someone they recognize who was more local rather than our team even though we had done some community consultation, gone out to communities, it was really a decision that this would be the most effective way, on both sides of the party, so in terms of us actually getting the recruitment numbers we needed and also for the community to really understand what the project was about. I was also involved in the design and the idea of the consent for flipchart. And that really was a recommendation from Researcher's learning about where they had done things, um done recruitment in the Northern Territory, and quite a common thing that Menzies Institute has used and something quite new to Perth though I think which was nice.

Interviewer: How long did the process for flipchart take?

That's a good a question. That's testing my memory. I feel like it was quite a--it was--it took--it did take a while--a while to design it more from the fact that we had come up with the initial flip chart and had a lot of team members review it, so it did go through the teams internally.

Interviewer: Yeah, um. There was a one day training workshop uh for Local Organisation staff in August in 2018. Did you attend?

No.

Interviewer: What do you think has worked well with the recruitment process?

What's worked well...Um there's been members of the Local Organisation team, for example [EHW name], who has been amazing, somewhat observations when I've been out on trips with her, where she's definitely got existing connections with some of the communities. For her to be able to look at names in school enrolment lists or even just names of community members as we've been collecting consents and be able to know, "Yeah, that person. I know where they live, I know who their family is, or that person is actually connected to this person." She has been effective in being able to really understand who is, and who to go to, her mannerisms as well and the way she can talk through the consent pro--so I did also hear her talking to once to a parent um and explaining the process, I think um. Yeah, so really some of the people that we train on the ground in Local Organisation have done an amazing job at actually completing the consent process and having that connection to communities that there is no way anyone in our team would've known.

Interviewer: So, this not the next question, but I'll ask it anyway. So in what way has working in partnership with Local Organisation influenced participation into the SToP Trial, do you think?

I would--I mean we don't have the direct com--comparison, obviously 'cause we um, we didn't do that much recruitment, internal members ourselves, but I do think um--I highly suspect that the numbers we got um have been quite good and they would not--we would not have got that amount of participation and that amount of people saying yes had we not had staff members from Local Organisation leading this process.

Interviewer: And there were different people in different communities doing recruiting?

Yes, so… there was a mixture of people and that was something that um Local Organisation guess coordinate and led and decided who were the best people um based on the training to go out and I do think that there was a difference between um who you know--if sometimes in some communities

Interviewer: And what were some of the challenges that um where [not sure if she says where] you encountered or the challenges for the recruitment process as a whole?

Um, yeah. [laughs]. There's a few different things. Some were like procedural, and some were just the nature of the beast, I guess. So by nature of the beast I mean things like i--it's tricky at times because there are not necessarily house numbers or not necessarily you don't know exactly who, definition of a parent or guardian can be different to the way an Aboriginal family compare defines a parent/guardian, so you know, who signs off on forms and dates of birth are not always known for children, and so some of that those items that are really pretty easy to fill out in research when you're not in these environments on a consent form and that kind of thing were much more difficult. Everyone did a great job at getting as much information as possible. Um, but that's I guess the nature of working in this context, whereas some of the other things that were uh tricky I guess was not um [pause] I have to think about how to word some of these things, um not necessarily having our staff--some--like being involved directly in the recruitment process, so not--we didn't often go out with the Local Organisation staff as well.

Interviewer: So geographically, it's a challenge?

Geographically, of course [laughs] it's a challenge. Um, and yeah, it means you can't just walk down the hallway and double, check in with someone. And geographically as well, then it's challenge in terms of budget and cost and I think we had originally set up um on the premise that the Local Organisation staff would go out once, to each community and you know, do that recruitment process, but realistically, in terms of certain times of the day, people not being there or not a good time to talk to people or whatever which Local Organisation really were good at making sure.

Interviewer: Did you consent any families yourself?

No, I didn't; I only did like a media consent form for someone, but I did go out with [researcher name] at one point trying to find someone to consent.

Interviewer: And did you see the um flip chart being used at any time during the recruiting

Yeah, I saw um actually saw [researcher name] using it

Interviewer: So, thinking about the challenges, um, what strategies do you think might help overcome these challenges for future research projects, particularly in the context of remote Aboriginal communities?

Um, I think it would be useful, if possible, to have like those communities as well especially where we don't have strong connections or health service doesn't have strong connections to, having someone--and upskilling people locally on the ground or finding someone who actually lives in that community would probably be um really helpful.

Interviewer: So, thank you. Um, so thanks for talking to me.

## Participant 3

Interviewer: I'm starting the recorder, so um on behalf of Telethon kids, thank you for taking the time to meet with me today to discuss um my recruitment evaluation project, um. This interview should take at least thirty minutes, and if at any time you would like to stop the interview, please let me know, and you don't have to answer any questions that you are not comfortable with. As you have consented, this interview will be audio recorded. All information will be de-identified, and you will remain anonymous. And I have started the recorder. So, do you have any questions before we start?

Nope, fire away.

Interviewer: Yup, and when you say um you haven't really done the consented, but you have, so can you just um, explain --if you have consented a family and if so, did you use the flip chart at the time?

Uh, yes, so I used the flip chart, um, it's so --I think I've only actually done one consent form um to be honest. Um, so yes, I did use the flip chart, um and it was actually with myself and uh Researcher, so he did the main part of the actual consent--he did all of the talking, and then I just filled the gaps, and the reason being is that um just 'cause it's more appropriate for him to sort of be doing it and explaining it. Yeah. Um just trying to think of what--'cause there was a lot of things we could have done better, so I'm just trying to sort of go, "What did I really like, and what could we have done a bit better?"

Interviewer: So that kind of leads on to --it's not the next question, but I'll go to this question. In what way has working in partnership with Local Organisation influenced participation into the SToP Trial?

Ooh, um, I think in some places, it has meant--it was almost 100% if not more than 100% people enrolled in the trial. Um, in other places, it meant of uh lower numbers [laughs]. Um, I honestly, I wouldn't know how it would looked different if we had tried to do the consent ourselves. I think we would have got lower recruitment numbers overall, um if we did it ourselves, but we probably would've been able to recruit and then screen on the spot.

Interviewer: So just for having um--do you think that's because of the connections that Local Organisation have in the communities?

I think so, yeah, I think where there was a person leading it, who was connected to the community, the consent rates were much higher. I think what they reported anecdotally is it was much easier to communicate the trial, the ideas of the trial to people um. Because of we've got so many different cultural sort of groups, so within the SToP trial surveillance, I think different people delivering the consent, so I don't know how to explain it, I guess having so many clusters and we--there wasn't necessarily people delivering--like, doing consent who had lived or connected to the communities in which they were s--like, seeking consent, um and in those communities, the rates of consent into the SToP trial were much lower. Um, whereas they were much higher, and they got through them a lot faster in the communities where the person collecting consent was actually from or connected to that community.

Interviewer: Okay, and what are some of the challenges with the recruitment process, either that you've encountered or that you know from your involvement in that?

I think both [EHW name] & I were the ones who were doing the bulk of the work on the ground, it was such a massive learning curve for the two of us. Things fell through the cracks. So we didn't have like good systems in place for how we're going to get the consent forms, what we're going to do with them when we've got them, how will we gonna identify you know the houses that had already been to, like I think the Local Organisation would spend an hour with each household, however, we had no way of accounting for when they went to a household and there was no one there, and how many times they had go back and visit that household before they said, "No, we're not going to get that consent." I think yeah, so there was a--and we didn't really know how to manage that and it was also hard to know like who had actually already said no, um and with the people moving around and population--um, movement, yeah, trying to figure out who had said nowhere--and where and um, yeah like it certainly wasn't a just walk up at a house once and expect there to be a whole family there who was happy to sign over to the SToP Trial, like they definitely had to do a lot of visits and it was a lot more work...

Interviewer: So, you're saying that it's a lot more time-consuming than they--when everyone initially thought?

I think so, yeah, and that--the thing is, I--I say that um from what I've been told anecdotally. Um, yeah. Um, what else was there?

Interviewer: You can take your time and have a think about thin--some things.

Yeah, um so who's [?]--yeah, could you ask that question again please?

Yeah, so what were the challenges you encountered during your involvement with the recruitment process or just broader I guess of challenges you know that occurred?

Yeah, um I think one--another challenge that comes to mind was the um things like attention to detail, um it was rather difficult to get consent forms back and find that information had not been completed and trying to figure out if it was human error or if that information didn't exist, so something like, you know, where a date of birth was missing, so we know that in a lot of communities, it's not uncommon for people to not know their date of birth, um but then there's also times where it was just human error, and you know information was missed. another challenge not having student lists or knowing where people lived in the community. Um, so I know that um I think Local Organisation--they had said they found it really difficult when they were um going around and they'd walk up at a house, and there was no actual children living in that house.

Interviewer: And what about do you think with the context of trying to recruit people like in the remote context um the challenges that come with that?

Geographically and stuff like that? Yeah, it's definitely [laughs], geography of it is logistically very challenging, very expensive, so I think when that challenge of I think for them to go out and go door-to-door, and find that you know, families aren't there, they've got to go back, how--and like, figuring out how many times they go back, and it certainly has an emotional toll as well and physically, it's actually quite demanding to be doing that. Just those really practical things.

Interviewer: That's fine, we can just come back if you do, if you want to jot things down, so from thinking about the challenges, what strategies do you think might help overcome these challenges for future research projects, particularly in this context?

I think ensuring people have the right skills I think we put a lot of trust into these guys doing the consent that they would be able to do it correctly and well, and they did a fantastic job. I think making sure people have got the right skills in terms of research and knowing and really understanding what good clinical practice is and means and I think knowing more about the trial to be able to respond more to those questions,

Interviewer: Do you think that if TKI and Local Organisation had gone out together, do you think that some of that would have been easier?

I think so, yeah, I think um, I think there needs to be sort of a--a bit of a line drawn in the sand of who has the expertise in communicating with the community and working with community and sharing information within the community, but there also needs to be more of who's got the expertise in either looking at skin health or understanding the research project um yeah.

Interviewer: Any other strategies that you think might help future research?

I think that Organisation Name actually has a really strong role to play in doing consent in the future. I think they were an underutilized resource in this process.

Interviewer: Cool, so what would you do differently about recruitment next time if you were to be involved in recruiting for research?

I think we got the idea right, not having the actual research team doing it ourselves, I think having that little bit of separation I think maybe we weren't--we separated a bit too far though. I think using Organisation Name would probably be more effective, I think probably how we really should have done is probably getting more involved in actually providing information to the community doing like a community events and yeah, actually going and sort of meeting people and answering any questions and concerns and then getting your community navigators and community researchers to go and go do the door-to-door thing um sort of straight away, and do it really, really targeted probably would've done it a whole lot closer to the actual time of the trial starting been a huge gap...

## Participant 4

Interviewer: Have you done any consenting yourself with the families?

Yes, so um when I've been out in I think in the East Kimberley, but also in the West Kimberley if there's been families who've come along to the school who have not um given permission for their kids to be involved, either 'cause they weren't asked or the form got lost, or they forgot to return it, or a variation of um those things, have um done the consenting process.

Interviewer: And did you use the flip chart?

No. Um, predominantly because the discussions I didn't have the flip chart with me, and some of the families had already seen it previously, so didn't um--like, I did offer to go and get it, and they say, "Oh, actually, we--we've heard about it before, we're happy to be involved, just quickly tell us what's going on."

Interviewer: And had you--have you been witness to other people using the flip chart when they have been consenting?

Um, I think I've seen people carry it, I think I have--uh, I think I have seen people use it, but I'm not sure that I've witnessed the entire um discussion, it's probably been um just--brief, segments of it.

Interviewer: Yup, cool, um so the one-day educational workshop that was held for the Local Organisation workers when you mentioned they were--need training, um, did you attend that? Were you there for that?

No.

Interviewer: Um, even though you weren't there, can you comment on how effective it might have been for learning about skin infections, or?

Um, well I mean I think our team ran the day, our team has training in learning and education, so I think that the--and also had a really solid understanding of the trial by the time that was run, so I think it was probably um reasonably effective. They also used some videos that I've made along the way to um describe the trial and to make sure that it um--they could understand the big picture as well as the task that they will be employed to do.

Interviewer: Cool. What do you think worked well with the recruitment process?

Um, I think it worked uh really well that um the communities where someone knew um the person who was asking permission um that worked really well. Like, when there was a relationship in place already, um the understanding happened really quickly. I think a big change when we um I guess created the contract to pay Local Organisation for the consent process versus when it was actually delivered, we had initially budgeted one person doing it for all of um the nine sites, and uh it became clear that that was um a big burden on one person and that um a group of nine people--or eight people would do um the consenting as a group, and I think there was variability in um skills, in understanding, and in um delivery. So I think that um there were some amazing um people who were from my perspective, what I saw was that the documentation of consent. So, it was clear the forms were filled out correctly, um it was um evidence from a paper point of view on paper point of view that the consent process had happened, and I might be inclined to assume that because it's well-documented, it was well-delivered. And when it was poorly documented, it was poorly delivered. I don't know that for a fact. I think that there were the ability to keep on redirecting and reframing and improving the process happened throughout the twelve months. So, I think it got better and better and probably faster and faster. And in the last weeks in the first round of the trial, um in some communities, there was a huge amount of consenting that was happening on the ground during that time.

Interviewer: Yup. So just going--moving onto a question about Local Organisation, in what way has working in partnership with Local Organisation influenced um participation into the SToP trial do you think?

That--I mean, that's a great question, I don't know. Um, I think Local Organisation's a brand that's well-known in um the Kimberley as an environmental health service provider as well as um management of housing. And I think the Telethon Kids brand is quite well known. So I think it was probably effective to have those two together, I think what we didn't really appreciate was any form of branding that might be needed, so we didn't badge the people with a T-shirt that said that we're part of the SToP trial and we're aligned with Telethon Kids, so I think that it probably relied on the skills and talents of the individuals who were employed by Local Organisation much more so than the support that say either of the brands was able to give. [we] probably could learn from that that next time, I think we probably would have tried to brand not with a stamp, but you know, creating a T-shirt that was for the team who was delivering um the consent, which sort of enabled people to recognize that they were both Local Organisation and to TKI and that it's about the SToP Trial, and it would probably have been a health promotion activity in its own right.

Interviewer: Do you--do you think um having Local Organisation--having that TKI person accompany a Local Organisation person as a team--would that have been effective m--or more effective do you think? Or uh?

I don't know; we didn't do it that way, so it's hard to say it, I have an understanding, but to be more effective or less effective. I honestly think it may have improved the integration between TKI and Local Organisation, and I also think it would have provided a richness for the research team to have been involved in that process, that we missed that opportunity, so I think having our team going house-to-house and doing the consent process builds their understanding of community, it builds their connection with community, and their relationships, so we lost that by outsourcing, I think we gain a whole lot more, and I think we probably um respected communities in the way that they were asking to be respected by doing it the way we did, but I think from our team's perspective, we did miss those opportunities to grow in confidence um, talking with Aboriginal mums and dads, and being present in community, and potentially even identifying champions within each community that would be great to work with in the future as part of the SToP trial.

Interviewer: What were the challenges that you encountered or that you know happened during the recruitment process?

Um, I think a really big challenge was um the one of privacy and around um the availability of school lists and actually any knowledge of who is in the community and how to find them. So, um I think Local Organisation had more ability to access that information we were probably to under say, privacy legislation as Telethon Kids, but it really did become clear that it was a really difficult process to track. And recordkeeping, I think, was probably poorly done, partly because of a misunderstanding that recordkeeping--records weren't supposed to be kept. Um, and partly because this concern around the privacy, and so you know, as well as many communities don't have a house map per se so that you can document on a map or something visual, where-where houses have been--already been visited and declined or um consented, so I think it made us very aware of the grey zones and it was tricky to know when we'd, saturated consent process.

Interviewer: In so, with the recordkeeping--sorry, it is about going to house and then having to return to the house and not knowing how many times, yeah

Yeah, I think that was probably challenging for all of the Local Organisation staff as well our own staff, it's that we just weren't sure you know had people been asked and declined or had they never been asked or had they been visited and weren't home, or--like, that sort of thing, and we probably could have done better with documentation. I think Local Organisation even reflected on that saying, "Well, that's how we would normally do business but for some reason, because it was research, we didn't do our business that way." And so, I think we probably all learned that process as much as the dissipation is really important, and to pay attention to those processes. I think we probably need to figure out a way forward whole of community-based consent to be able to understand whether everyone has been asked and declined or not asked at all and that sort of thing. So, I think you know, if you're thinking about a mass drug administration, where you might want to um involve everyone in the whole community and research trial, we could learn a lot in and to implement that in the future.

Interviewer: That's the next question. So, thinking of these challenges, what strategies would work well in the future for recruiting research projects, particularly in this context?

Um, I think we probably would have met more regularly um with the Local Organisation stakeholders, and probably I think that to name names, [EHW name] and [staff name] I think met up regularly, potentially as oversight of the trial myself and [Name] ’s oversight of Local Organisation probably didn't meet as regularly to just touch base and see how the process was going. We probably trusted each other to just do it, which I think is a really important thing to do, um and to let it be. But I also think we probably learned along the way we could have improved things sooner had we been paying a little bit more attention to it, I think that the--I think involving--I think we talked about branding, and I think that’s quite important. I also think that the use of our research team alongside the Local Organisation team--not always, but would really have strengthened partnerships, would have um given greater cultural understanding and that opportunity to get to know community members who could potentially be future parts of the SToP Trial team. Um, I also think that because it took twelve months for the consent to happen, there's so much information and understanding that gets lost over time, and so was there a way that we could have done it faster, should we have thought about having more people do it, more quickly and um just cracked on and got the study up and going, how do we ensure that the consent that we had given um say in 2018 is still valid in 2021, and I guess the other thing we learnt was that consent was acquired for all of the children regardless if they're at school yet because we knew that they'd eventually get to school, and so that left us with a lot of consents that we had, but we weren't sure whether they were actually at school yet or if they were in high school and away, and so there was a lot of uncertainties, so probably thinking about our forms and how we could improve our documentation, I think would probably be the other improvement I would make.

Interviewer: Because--did the parents or the carers, did they get the option to consent for some things and no other things?

Oh, that as well. So I haven't even touched on that is that we did do a layered approach to consent, and so I think that's really important for information, so that the family is aware that there are multiple activities that will occur, having a multi-layered consent did alert us to concerns in one community where asking to check weight and height on children, worried the caregivers that we might be using that information to inform the--I guess, the DCP and that their children were malnourished or something like that whereas that information was being collected purely from a dose--drug-dosing perspective, it wasn't needed for any other reason and would never be used for any other reason, but that disconnect between the research team and the service provider doing the consent meant that wasn't clearly understood and made it difficult. So I think that the consent is very much akin to a sales pitch, and not because you're trying to pull the wool over someone's eyes, but the enthusiasm that you show for the study and the interest and knowledge you have for it, in the same way as a salesperson has about trying to sell you a car, you're--you definitely much more engaged in that if someone knows their stuff, and so if p--people don't know their stuff, it can often be um quite tricky, from that perspective. I think if we did it again, we'd probably still use that layered approach, but we probably um maybe think more strategically about how much information is provided and what are the elements that need to be on that that parents need to give permission for and um, what can be implied or inferred um and that sort of thing.

Interviewer: Cause that's the next question. What would you do differently about recruitment next time if you were to be involved in recruiting in this context?

Um, I think we've talked through most of that, I knew--I would definitely um use this process again, I think we can learn from it, and I think we could improve it dramatically, but I do think that the wisdom of the partners to ask us to use people known to the communities to go to their homes and ask for permission to be in the study, is a principle that I 110% support. So would do it the same, I hope that when we get to do the next study, we will have um a much richer web of knowledge and engagement with the communities that there are people in place who we can readily employ to do this kind of work, which is good for them because it's employment and it's capacity-building, and it's involved in informing information about their families, but also great for us because it means that we're doing things the right way, that our business is asking for consent in a way that is acceptable and appreciated and feels connected and doesn't feel like that sort of helicopter fly in, fly out um, you're taking our data away from us, um feel , which um unfortunately research has, and I don't know that we'll ever get away from that completely, but I think by embedding it in partnerships, I think we can probably do a lot better.

Interviewer: Okay, so that's the end of the questions, and if you don't have anything else, that's all good. I will turn--I think we're at twenty-five minutes. I'm just gonna turn the recorder off now. Thank you.

## Participant 5

Interviewer: So, on behalf of Telethon Kids, thank you for taking the time to meet with me today, and discuss um the recruitment process. It should take about thirty minutes. If any time you would like to stop the interview, um please let me know. You don't have to answer any questions, but if you have questions for me, please feel free. With your consent, this interview is being audio recorded, and all the information will be de-identified and you will remain anonymous.

Great, okay.

Interviewer: Do you think have any questions?

Um, no. No. Not yet, so [?].

Interviewer: Okay, cool. Um, last year in August, there was an educational workshop for um the Local Organisation staff. And it is my understanding that you attended? Did you attend, yes?

Yes, so I did, yeah, yeah. So, I was giving the workshop um along with [Staff Name]. So pretty much um yeah, we uh--we knew that Local Organisation were going to do this job, and then it was initially supposed to be one person, but then Name decided it would be multiple people, and that she sort of divided the job out between all these people and again [laughs], it was a pretty rushed job, it was sort of like, "Right, let's--we've got to do the trainings so we have to get together sort of a training outline," which really kind of touched upon getting into GCP--do you want me to talk about that now or do you--have you any other questions?

Interviewer: Yeah, no, you um you go and chat about that.

Okay, as you know probably the staff in Local Organisation wouldn't have much training in research, and they wouldn't you know be too familiar really what it's all about very surface kind of understanding. Because they're just not obviously in that forte, or they weren't anywhere at that time, so we certainly felt, um, "Right, we have to give them a really in-depth rundown of the SToP Trial um so that they know what they're asking people to consent about, so we need to kind of go through all the details of the SToP Trial." And I--uh straightaway, I remember thinking, "You know, it's taken me quite a half a while to get my head around this," so it's, you know, it was trying to design the training so it's very easy to understand, but also making sure you hit all the important points. So, and also then we had to kind of give them a bit of background on what research was, what GCP was, where GCP had originated, what kind of things had happened in the world of research historically to kind of meet GCP, an integral part of anyone who's trying out research, understanding why people would have issues with no informed consent, and all that kind of stuff, so we sort of put a story together based on the GCP program that's on the we actually do ourselves, so we kind of amp up a bit--a bit more interesting W--World War II pictures and thalidomide and things that kind of, I suppose are easier visually to understand that "Look, this is what can go wrong if you don't um you know, if it's not ethical how your research is worked out and stuff." So that was what it was, then basically,

Interviewer: Ah, cool. So, what do you think worked well with the recruitment process?

Um, I mean what the numbers in the enrolment tree that we have, I can't remember off the top of my head, but look, to be quite honest, like if it was me that was trying to go out there and get people signed up for this trial, we wouldn't have probably a third of the numbers, um and like I know from [staff name's time] in the Kimberley doing that sort of work for a long time, she had kind of reiterated that, that there was no way people would sign up, they would just kind of say, "Oh, come back later," type of thing, whereas I think with [EHW name[ and [Name] , they were able to say, "Ah, look, no." [?] Even if they didn't know people, 'cause I think we had a bit of a misconception that they were in community a little bit more than they, are just said people that were in community were actually from that community, whereas that’s actually not necessarily the case when we came out the other side. It was sort of like, they're all Local Organisation staff, they’re all Aboriginal s--staff, but they're not necessarily when the person went to [community name], they didn't necessarily have any links to [community name].

Interviewer: what worked well, yes.

So I think what worked well was the fact that we got a lot consents, not because I think you know, in some communities, well over a hundred percent, so that worked well, I think um working with [EHW name] was great, she was with--she's like an up-and-coming Aboriginal health research, like, in you know, I would hope from this she would build on that and hopefully you know, forge a career in this area, what else worked well, I guess um yeah. I think that's it...

Interviewer: So, this is a question that's a bit, further down, but I'm gonna ask it now. So, in what way has working in partnership with Local Organisation influenced the participation into the SToP trial and you've basically answered that question, but if--is there anything else to add to that?

I think you just gave it that kind of credibility and it's sort of showed community members that we were serious about um capacity-building Aboriginal people, and not just there as I said "Walk the walk," but actually--oh, sorry "talk the talk, walk the walk," and I feel like it’s the first step sort of before you get to actually training up community members, which would be a next step probably in that process, but um so I think it gave us credibility, it also gave us numbers we wouldn't have got those numbers absolutely no way Jose without Local Organisation's help. And it gave us well because it wouldn't have been the right thing to do for us just to walk up in the community and start asking people 'cause i--it would have been sort of--it's such a complicated trial, I think that's the big thing I'm trying to get at in a very roundabout way, I think it's such a complicated trial, it's not very easy you're not just saying, "I just--we just want to take a picture of skin sore. We're kind of saying, "Ca we have your swab for future use?" because we're looking it down the Group A strep path down the line. We're also there's the element of you know, taking skin swabs, taking heights, weights, all of--like, there's so many different elements you're asking for consent for people have issues with some of them, and they don't want to sound maybe it's only a simple thing, but they take it might be sort of a bit more, something behind it. So, I think you needed somebody who understood it well in their way to be able to understand--to be able to relate it back to people in a culturally appropriate way. Yeah.

Yeah, yeah, definitely. Um, so you talked about what you would do differently next time with the recruitment as clearer processes for paperwork, so um thinking about the challenges, what strategies do you think help overcome these challenges for future research projects?

Look, I think a sort of um uh--eh thing that could--the job that needs to be done a month before we go out would be a really good idea because if I leave--

Interviewer: So closer, closer to the time that you're actually gonna start, is that what you're meaning?

...we went on our first trip in um in May, right? I feel like if we had said to Local Organisation, "Could you please be finished by the 29th of March?" Um, all our paperwork--most of our paperwork could have been kind of done by then, and also sort of the idea of what numbers we had and stuff rather than literally kind of were still getting paperwork the day we were going out, and then that's the other thing, right? So, like probably know this as much as I do. When we were out there, we were then getting recruited--people were being recruited.

Interviewer: Um, alright, so do you have any other further comments that you would like to share about your experience of the recruitment process that I haven't covered in my questions?

Um, let me think

Interviewer: Yeah, take a minute to have a--have a think.

So, we talked about sort workflow issues, I probably would call them workflow issues of timely getting back consent forms and then um sort of management of doubling up of um people--oh you know one other thing I really think is like, you know, if you were doing it again, you might even get the people who had consented to input the data into the database. You know, 'cause then it would be very--it would be much more--'cause they know who they saw, and they know--and the doubling up and all that will be less likely to happen but that anyway--that's just kind of an aside. Um, so we talked about workflow process, um I think it probably another thing to mention was the fact that there was often cultural reasons why consent couldn't happen, and because of that, you really need to look at a big picture, and have a wide berth of ideas about when you can go and like have like, yeah, don't leave it to the last minute again because there's often a funeral, there's often sorry business, there's often logistical reasons you can't go to community...

Interviewer: No, definitely not. Well, thank you. I'm going to turn the recorder off now.

## Participant 8

Interviewer 2: So, we've turned the recorder on.

Interviewer 1:OK so I won't say your name OK um is there a number I need to use? No OK. Just going back to the first question as we're doing a lot of skin health stuff with this particular interview or this particular promotion what we want to do is ask you about community life, skin infections or health in general. So, what has happened since our skin has come onto the scene, what, what do your um can you tell about someone coming to talk to you about skin and who were they? Where were they from?

Yeah think it was Local Organisation the girls came from Local Organisation they was having a chat to me about the sores skin and sores at school like sort of trying to get a program happening but with the school they was discussing you know different sores and stuff and medications and you whether you use creams and antibiotics yeah so just but I knew most of all of that anyway because I have children myself & regularly taking them to the clinic to yeah get them checked out

Interviewer 1: And they explained the antibiotics, one was a

Yeah, like a topical like a cream like you put on your sores and other ones might need antibiotics to help from the inside. So, the difference between the two, yeah so medication

Interviewer 1: So, do you recall, you don't recall this chart at all? Do you

Nah not the not that particular chart but (rustling sound) I think they had the paper.

Interviewer: OK, to get to get you to sign, to ask you to sign

Yeah

Interviewer 1: So, you remember them explaining the cream compared to oral (P agrees) taking a tablet...OK and the person who came to you was it an Aboriginal person?

Yeah, it was an Aboriginal person

Interviewer 1: Do you know, do you know who it was

Yeah, one of the [names female]

Interviewer 1: Right, one of the [names female]

Yeah, with Local Organisation yep

Interviewer 1: And you found that um she explained to you what the study was all about

yeah, explaining you know in ways you know that would be easier to understand you know and just breaking it down what they were there for and what they was trying to like um get information on yeah stuff to do with

Interviewer 1: And the way she explained it

Yeah, it was you know way you know that I can you know like it wasn't hard for me to understand so she used words like you know Kriol to you know as well being able to interpret or even yeah

Interviewer 1: And did you find that different from a non-Aboriginal person or a doctor explaining it to you

Yeah like you know with the doctors you know like they tend to make you assume you know what they're talking about and using the terms and way of speaking which is different to you know the way you know we probably grew up hearing you know or having something explained to us you know and yeah so sometimes we don't know the terminology or you know the language that they're speaking and stuff yeah (conversation in the background)

Interviewer 1: So apart from you learning about or already having some idea about skin sores and their do you understand the effects of skin sores if it gets worse

Yeah, if gets worse you know you get Rheumatic you know like heart if untreated, kidney like internal organs I think and be affected by it & having an open wound & not treating it you know like properly in a in short amount of time um yeah and just having the right medication to use and then identify what sort of sore it is you know... yeah

Interviewer 1: So, what about scabies, you know a bit more about scabies

Yeah, like they're little insects that can you know burrow deep into your like your pores of your skin and that create sores and boils and blisters and yeah around the joints, the fingers, and the hands you normally need a cream to…

Interviewer 1: To treat it

To treat it yeah

Interviewer 1: What about (Aboriginal word for lice) lice and all that

Yeah

Interviewer 1: What sort of did they explain anyone talk about stuff like that to you?

Um nah, not that not so much the lice but um

Interviewer 1: More they just spoke about the scabies mite

Yeah, the scabies and the

Interviewer 1: OK wonderful, thanks um. So, what would you do differently, do you from your knowledge and you're saying you had a little bit of an idea about it

Yep

Interviewer 1: And from your knowledge have you spoken to any of your sisters or brothers or family members and if you were or had to how would you explain it to them? Yeah, about this

Or about the sores and stuff

Interviewer 1: And about what we're doing

Yeah, um Yeah, I'd recommend them having you know like work with the schools you know, sorry what

Interviewer 1: Yeah, work with the schools…Yeah, work with the schools to

Interviewer 1: To get their kids

To get their kids to get like you know taking notice of the children you know monitor like not so much monitoring them but like keeping an eye on so that maybe that they like after a few weeks you know and then have them reminded you know like the parents sometimes we can forget you know yeah

Interviewer 1: So, if you see a pussy sore now what you would do if you saw my kid with a pussy sore would you

Yeah I'd recommend like you know taking them to the clinic and get them you know to have a look at cause I always tell em sometimes sores we have you know like open wounds and stuff you know can if they need to take them to the clinic to help them you know like treated or cleaned so it minimise there like having rheumatic heart, fever and kidney disease and any bacterial that can get on the inside through the sore

Interviewer 1: mmmm

Yeah, be able to get the right thing like medication they need and yeah

Interviewer 1: So, you'd have no problems coming up to say someone like me if my kid had or some like you sister's kid or even a community member you'd have no problems going to them and talking to them about that

Yeah, you know like especially family because they close to us you know and I'd feel more comfortable than someone and I also think

Interviewer 1: Yeah so, you'd feel more comfortable talking to family first

Yeah, to family you know first before like not just telling anyone the sore is not thing you know

Interviewer 1: Why would that be? why would you not tell anyone else

I think you know like I think it’s the conflict that might arise too you know like might get like defensive and start growling or you know like being told (F1 yeah)

Interviewer 1: So, what's so what's the best way to do it like Local Organisation come and do this is there any other way you can see this sort of stuff thing being done

I reckon like maybe in the schools or having something like that in the school like

Interviewer 1: We're doing it now in the school do you see any other ways that to really get a really strongly embedded into the community you know to really get it so that the community people are not just like your family learn but extended family and our all our other mob learn as well. What's can you see any other way of doing it we can do it at the school we can do it at the clinic we can talk about it in front here but is there any other way we could do it do you think to spread the word about how to treat scabies how to prevent stuff the environment at home you can stop you know skin infections by showering regularly by when every time we go out and play in and before we eat and do other stuff washing our hands that we're cleaning up can you see any other way that researchers can do this type of um study to help our mob get better and healthier so then they can then end up treating themselves

Well i think like have someone coming around and seeing them you know and like talking to them about stuff like that they wouldn't probably take notice as much being at the clinic or the school apart from having maybe brochures and you know stuff like that having I think just getting the word educating everyone more you know so they know like what type of sickness sores and stuff can happen I think just some awareness maybe you know like you know having the knowledge probably like at home starting at home yeah

Interviewer 1: OK. So, we had (Local Organisation staff) come and do it can you see anyone in [Community Name] doing something like this like (name) has done

Yeah

Interviewer 1: Not from the school not from the clinic can you see anyone else

Like within the community?

Interviewer 1: Yeah

Yeah, like I can see that like you know happening yeah like with um like the community members themselves maybe or like in the community like having a like um

Interviewer 1: Who would you target in the community - Ok you're living in [community name] but who would you target here in [community name] to get the message across?

Who would I talk to

Interviewer 1: Who would you think would be a good advocate .name someone like family do you think. Who's in a position in the community to do something like to be like another (Local Organisation staff) another Local Organisation person, not working for Local Organisation but outside of Local Organisation . Could you see someone like (community member name) doing something like this?

Interviewer 1: Would they listen you reckon

Maybe they might

Interviewer 1: What about the council would the councillors be more proactive in this space? You know with the council getting involved do you think they'd listen to the councillors?

Yeah, they'd...like

Interviewer 2: Can I just ask one last question. How, what do you think um how do think research should be conducted in community and trying to engage community into research so people/communities are involved right from the start of research. How do you think that should happen?

People like in the community to go along with the you know like working like work alongside the community members like having someone from the community go around maybe with them

That would make them more comfortable to talk maybe and...rather than someone else coming in

Interviewer 2: Rather than just someone like me coming

Random you know (laughing) Yeah, but I reckon working with the community, having people in the community do that I think

Interviewer: Yep, awesome. Do you have any other questions, or would you like to make any other comments about what we're doing um as far as the skin the skin health is concerned or

Nah but you know like to see more and more of our people you know in the medical industry like you know like....sector having nurses and doctors and that that are Aboriginal people you know. We're gotta be proud a bit of self-determination there you know and be happy about it see someone else doing something good with their lives and

Interviewer: Yep, awesome. Thank you so much for your time um I'm gonna turn the recorder off now so (recorder switched off)

## Participant 9

Interviewer 1: The questions I'm going to ask you about is um really about knowledge, attitudes, and practices in community and whether we've aahhh gone about it the right way um what we have done is ask Local Organisation [staff name] who went around and recruited parents and asked parents if they wanted their kids surveyed into the skin health stuff. So yeah, what was your thoughts on Local Organisation doing that. Do you have any thoughts around that. Can you see it being done differently or is what they way (name) did it was great or fine or.

Yeah, they come into the school and present something that the kids and some of the parents were there, so they were aware that it was gonna happen in the community. Some people they just rock up and they want to do all of these things but no notification or information to um to these kind of things. It was really good.

Interviewer 1: So, using Local Organisation you feel that it was culturally appropriate in the recruiting process, could you see advantages in doing it differently?

Nah because they came, they come knocking on the door give us information about it and um asked us if yeah like in the paper they ask us if we wanted to do it be in the trial or not to be either way...yeah. But they wasn't pushy or anything, some people when they see like especially in communities when they see non-Aboriginal people rock up to the door, they just want to shut the door and go back inside they don't want to talk but it's good for Aboriginal like Local Organisation people to come out and talk to cause we all we know some of the Local Organisation workers to the countrymen’s you know…

Interviewer 2: Yeah

Interviewer 1: Did local people give Local Organisation much help in finding the people and doing all of that getting them onboard like you say you knew the Local Organisation people

Yeah, it was yeah two boys that one used to live here, they Bardi boys, two Bardi boys themselves, yeah and another in-law family

Interviewer 1: Oh, so they used people from

No, they was they moved into town and the family (name's) family are the main ones they moved but they got sent out to do the surveys and stuff like that

Interviewer 1: Oh, so Local Organisation recruited them to come, (name) with us

Yeah, they came one day one of the boys come there or (name) come there and yeah asked me showed me the quickly showed me what it's about and if it was OK

Interviewer 1: But it was (male name) oh yeah OK awesome. Cause they lived here so that was the advantage that (female name) had. Um the flipchart, did you find this really easy and simple and self-explanatory the way they when they went through it and you know when they talked about the old or the current way of medicating for scabies and the new way they are thinking of doing it. Was that did you find that easy? This explanation now, who's that one you look after that daughter one that had rheumatic heart

Yeah that's (female name)

Interviewer 1: Yeah (female name) yeah like she gets the needles ay

Yep

Interviewer 1: Yeah, so now they're trying to look at the current way of doing it and also this way

Yeah

Interviewer 1: Um, not that (female name) will get it this way but anyone new getting into the program will use the new way. (P, yeah) of treating

Yeah, now that's good

Interviewer 1: So, you found this simple and easy to understand (points to flipchart) but probably your knowledge was already there about all that stuff. What about, yeah go on don't let me stop you, I'm rushing because I know you've got to go um don't if I’m rushing just pull me back. What are you seeing any difference in kids with sores and stuff like that coming into the school, I mean 0-3 program

Well, because the 0-3 program was only this our 4th year yeah no I've soon as someone comes in as the coordinator I try and get like straight onto it and whoever comes in and want to talk about early childhood stuff or children's health and things so like um yeah, I'm aware and those kids

Interviewer 1: OK so was it because of this that now that you are much more proactive in doing that do you think that this sort of value added to you saying look, we need this trial so it's been since the introduction of this flipchart and the team coming in and talking about the skin sores and rheumatic heart

And make more difference and taught more parents to be aware and go straight into, not just letting it be you know, with some of the sores the go oh it'll go away and as soon as them see this they go straight

Interviewer 1: This made a difference, this flipchart, and the team and yeah, OK, um I don't have anything else so do you can you still see is there another way of doing this, we've used Local Organisation um is there any other way you can see that we could do this to recruit people on community.

Interviewer 2: So when researchers come in um instead of like you said they just come in and nobody knows what they're doing what could be a way to keep make sure that the community always aware of what's happening when researchers come in and so um how could we do it better or if we could do it better next time

Interviewer 1: And just to add onto what [researcher name] has just saying is that not so much the awareness but how can we get provide an education for the people themselves to know what to do you know like um you get [researcher name] will come out to the community and want to do something or where does she start and who does she start with

Well the first place now that the community navigator stuff like what is it the family, that’ll be a good start you know you gets some of us well I was vice chair last year I'm still on the council again so I spread it out so it's very so everybody knows about it but like even if cause then now the community navigators are doing stuff you know like all kind of health stuff and learning and focusing on early childhood and kids and ages from 0-3 think 12 or 17 so I think it be good to start their cause then she goes to everybody like the three main navigators they go to every single family each family groups and talks to them and that'll be the best way to do it yeah.

Interviewer 2: Awesome

Interviewer 1: So, using people specifically from the community that live there to go out and contact cause they already got the contacts with them all the families and to recruit them to get involved in research

Interviewer 2: Awesome

Another one would be like letting our CEOs know and we can actually have special meetings you know like invite everybody to come to the hall to explain like stead of going knocking on each door you'll get em all in one group you can have 60 something people 70 people right there and instead of doing house to house option it takes 2 or 3 days to do that when you can do it in 4 hours yeah. So that's two good strategies, I think I know that doing it individually will take time when you go knocking door to door but that way, you're really inclusive but I also like the idea of doing in a community in a whole getting everyone, so I think that's a good idea, mmmm

Interviewer 2: Awesome, have you got anything else that you'd like to add or suggest cause it's really important to get your per to get like your opinion and your way that the community would like to see it being done so thank you for speaking with us I'm going to turn the recorder off now just so....(recorder switched off).

## Participants 10 & 11

Interviewer 1: So, we get you because of what's happened to you (Interviewer 2: I've turned the recorder on) so it's a great opportunity to speak with you (Interviewer 2: Yeah, very yeah thank you for speaking to us)

Yeah, we went through that chart, we were all sitting around most of us were mothers and there were other people like (community member name) and them were involved in that meeting that recognised things um even us mob and then some of the mob that work at the schools

Interviewer 1: Cause um yeah cause if there's any trauma to the skin can allow that particular bacteria to get into the blood system and that's what’s happened

And I've found and even now even I work in the school before this job and people now would like my daughter works in pre-primary and stuff and daughter-in-law and things and they say like you know they see kids with skin sores and that they said they would send them home and the little process there you know like but it's not for us it's about that parent taking this child to the clinic is the child being treated so that's the break down thing I think I see I don't think you know like there needs to be a follow-up with certain that child is being treated...

Interviewer 1: And you finding the same with those concerns as well?

And even just um even as parents like when the kids were little the lack of education with the kids you'd be home or the kids are sick people's kids are sick and they're not going to school today or whatever or they have sick babies or sores and ringworms and whatever and they come over and visit you (laughing) oh my god my kids gonna get it now because (talking in the background) and you know you're not trying to be nasty but you have to go home with your child your child is sick here so they all end up with ringworm or you know scabies or whatever you know

Yeah, or gastro goes the same with everything it's like oh I'll go visit this mob and we got a house full of kids, so I mean you know

Interviewer 1: So that's a good example

Cross contamination…Lack of education

Interviewer 1: So how do we improve that how do see

Well I think more parents and maybe it needs to start at the 0-3 we have a 0-3 program now so the mums actually have to come and be in there with them at this program so maybe it's a good time to grab them then but then you have the same parents coming in to bring the child so those ones who's sitting at home are those ones who likely need that are missing out so you still need to fill that gap of how do you reach them, do we have child health nurses going out there knocking on doors or people are reluctant as you can see we hold lots of stuff we have environmental health or whatever else, we have all these um things for people to come along the type of groups that are never coming that's where we losing out you know. My concerns are all these things those people that are sitting at home not getting this and a lot of it and we've talked to this about and it’s the same with everything from meetings to everything you know and these sort of things are happening it's usually those ones sitting at home that how do we get them more involved you know how do you know I mean they've got to take part and want to participate too or whatever but I'm thinking a lot of people feel more comfortable if you just go and do it at their house or you know also maybe that’s and maybe doing something with the whole family as one I’m not sure um

Interviewer 1: Do you think to do it that way then to recruit someone like Local Organisation to do it or is there someone else in the community or another organisation

I think the stigma I think seriously is out there cause I have a lot even with us when we do tenancy support and that kind of stuff, they still won't let them in and I talk to them at the door but I always get these calls and say can you come here because this mob so I go there and it's like - hey OK let's come in so you still need a local contact

Interviewer 1: From locals

From locals they think that we are scrutinising the way they live, or we are judging them or whatever, so they put up all these defences and barricades to say no right yeah OK and goodbye so still don't get to deliver what you have meant to or you're trying to do

Interviewer 1: So, what you're saying

You need a local

Interviewer 1: Yeah, you need someone so you are saying someone who come from the outside would always come to you and get you to get them through

Support them and even though these people even like one their officers is local one of them lives in the area but still they still say I can't get past their door (phone goes off, difficult to hear)... I have to go there and say you mob you need to go to oh yeah, no worries come there's a difference of attitudes when yeah it's approach but it's like when you're from the department or you're from an organisation they think they're judging me they you know they feel invaded and their privacy

Interviewer 2: So, you think people will feel more comfortable

If they had a local person there

Interviewer 2: If they know the person and they trust the person (P10. Yeah) OK

Interviewer 1: But not necessarily that even if they know the person, they come from an organisation your saying they still feel judged

Interviewer 2: So, it has to be a local community

You don't have someone from here go to [community name] or [community name] you need their own people to do it. So somebody like for example being a health thing would be our local health workers maybe you know be our point of the door knocking the education process of you know I don't know but that's what I'm just thinking for delivering like I found and not saying so much but I did hear after and just this even (Local Organisation name) mob they you know locals of [town name] and everything and we know them like I know them a lot of people was like oh they didn't come to the thing or you know and I gave out a few consent forms for her and people still was reluctant to actually participate in the program thinking that they might have been judging them or judging their children for not looking after their kids or whatever...

Interviewer 1: So, the only, so what I'm hearing from you (name) the only way that (Local Organisation name) could get into those that were she was trying to recruit was to come to someone like you

She got to link up with maybe (name at the clinic) you know what I mean

Interviewer 1: Yep (everyone talking at once)

When somebody give us

Interviewer 1: Yep, we know exactly what you mean and um

In the community that could be like you know a face to say they all right it's fine we can do this it's only about this we're not talking about how you mind your kids or we're just telling you give you this information

Interviewer 1: Awesome yeah

We here to help or not to judge you know cause that's the biggest with people all of us everybody put this thing up or they're judging us and then you know things like that people at all you know they want anyone knocking on their door but I found that even when I was sick in Perth and all my inspections that I do all the other time everyone's happy and yeah come in cause the other officers are around the three months I was in Perth see they couldn't get in the door they're ringing me they didn't want to let me in I had to ring them and go "let them in" you know so it was still the barriers so

Interviewer 1: so just quickly explain is your role the[role name] isn't it that's why the only way people can get in or get to see people is if they went through a local. So, I think we're getting what you're saying and it's that it is

It's fine to have a local on the ground with that expert or professional person that's coming to deliver because it makes little difference the types of groups you're going to be reaching

Interviewer 1: And even though (Local Organisation name) Aboriginal and was from Town name and all that didn't matter she doesn't live on community

Interviewer 1: That's really valuable (name) yeah no thank you so much

Interviewer 2: so, like researchers’ um and if someone wanted to come in community and do research in the future what you're how would that be done well how would you as a community member um want to see that done similar fashion with would you like to see things like flipcharts or what kind of things and what

These are good (pointing to flipchart) if you're like doing one on one stuff because visual is always good for us mob like that's really plain and simple people can relate to that oh we've seen that sore on our kid but we didn't know what it was oh you know it makes people think not too many words and stuff because I think this is sort of good for the um when you go to do the door knocking stuff and the one on one stuff you know and people might bring up concerns oh yeah so you know they don't realise all these sores here can affect your heart later on oh cause there's a lot of stigma around rheumatic heart that I've been finding these people think it's gonna run in your family like a heart condition or diabetes or whatever cause it's always been

Interviewer 1: That's it's hereditary rather than

Interviewer 1: So, the education and you see that people are learning more now or from what the skin trial is done and what

Well, I'm not sure like if everybody would know maybe the mob at the school because they'd have these kids coming in and you'd know if that education is there they try to encourage like the different stuff (f1, would you) or something

Interviewer 1: So, you're happy with that um so the only thing you see differently is that we should have someone locally doing this

On the ground you may reach more people

Interviewer 1: do you feel though because that I'll through a spanner in the works here because researchers only comes in and works only for 3 to 4 years at a time with are there people here be prepared to do something like that and go and work on a part-time basis which might be one day a week or one day a month or one day a term where there or one week a term where they might do the pre-visit um and let everyone know that Telethon is coming in and then get everything ready for the researcher and maybe later on um get feedback from parents on how it all went and provide it back to the researcher...

I reckon that would work yeah you know um as well but actually when like you say when the services are actually here and it's actually the critical time of having participation is more likely with that local person is needed to say look at this happening today (claps hands) we need to go yeah or whatever encourage them to come down or to go door knocking with them you know

Interviewer 2: So, when I'm just going to ask a question so when um Local Organisation came and spoke about the flipchart and things, they did they go they went door knocking and they also did they do a group session like a like an information session or was it

(Local Organisation staff) came in with us because we were actually having a meeting and then we said well come tell us what's this about

Interviewer 1: This is all the family group

Yeah so, this um and then she went into the school I believe from what they were saying (f2, yep)

Interviewer 1: Oh, ok so she did both ways...but (Local Organisation name) also helped her

Recorder Turned Off - Participant 11 left the room

## Participant 14

Interviewer 2: So, we got the recorder on now um, and did you want to start Researcher or?

Interviewer 1: Yeah, I'm happy to start, we what we're wanting to find out is the way our processes went and how we can improve them on it and do it better um you might have remembered Local Organisation came and did the consent process and it would've been (staff) just want to talk us through that. What your thoughts are?

At that time I wasn't um I wasn't involved in that but (staff) came up to me and said you know what she was doing after they was on their way out so I really didn't have anything to do with that process until later on you know because they was coming in to the community and just asking all the mothers about it and she came and seen me later and said we've done all this it was too late I didn't know through Local Organisation and Local Organisation do not I think if we want to Telethon to work more closer with them they need to be more um involved with the communities people that they're dealing with...

Interviewer 2: What would look like?

Yeah you know like um maybe through coming and giving us more updates through their organisation you know come to our community meetings at the moment this one happening people are just coming services just coming in doing their own business and going out again so with this Telethon stuff if Local Organisation is part of it we need to work more closer with them because their they will be more on the ground than Telethon is you know you come out four times a year...

Interviewer 1: How would you have done what has just transpired in terms of getting the consent ah consent processes done how would you what would you consider another way of doing it? Like you had (staff) come and do it um is there any what other methods would you use to make people understand what they um what they're signing up for

Um I'd have a little BBQ and just to maybe a little you know like a sign banner or something to say you know who yours are so put that up so people know you know like um a lot of people don't read in these communities are not read they don't listen they walk around like that sometimes (Interviewer 1: yeah) because of this it sort of needs to more advertised like (Interviewer 2: what's the best way to advertise) well we've got a FB page here at the school

Interviewer 1: so, you want more of an information sessions you want it like a community gathering um something outside the store or in that common area them having a stall and then explaining everything

Because we're the last community at the on the track we can tend to not um we sort of miss a lot of stuff unless we you know lucky, I'm here I'm vocal and (community member) we actually miss out on a lot of things

Interviewer 1: So, name would be another person worth talking to is she is the office now?

Yes, she would be

Interviewer 1: OK. So, what other ways can you see it working that would um make people understand what the study was about and why we were doing what we were doing and why you've given consent particularly why you've given consent

Well I'm sure there's people in this community they would welcome that stuff you know they'd welcome like to for them to be able something to come to you know with their kids um yeah I I find that there's too much talking when people come here more visual (Interviewer 2: ok) yeah more pictures more or maybe just chuck a DVD on or do a PP you know something else... you know there like ah you know little iPad or something you know where people can just if you got stuff on like you've got a page on your on the website or something tell people because it's a lot of technology now you could tell them all there's this you know if you go here you can find more information like that because young mothers they really um technology's going the way it's going you know

Interviewer 2: so, they would already be looking probably, and we can here's one you can look at

Mmmm and they'll look at it you know and then they'd start checking their kids sores oh right to go to hospital through technology even the kids are more technology than the mothers and that the babies and if they see something you know that like what I think more visual in these communities

Interviewer 1: When Local Organisation did the consent was there anyone locally going along with them, or they had direct access to everybody they can just go and people just invited them

I don't know I never got spoken to they might have spoken to (community member and cm) because (cm) another good person to interview was would be um (community member's husband) because he works with organisation name and like that's the um clean-up of the community and stuff like that so he could have his issues he might have issues around this like he could tell you that he has all these problems around resources again you know so if we want to improve it and we working together more ears hear it something might be done you know the rubbish truck or we don't even have a rubbish truck have you seen us how we collect our rubbish...with a trailer and a tractor you know those they might sound they sound big when you're living out on a community because it's s in town and you don't have to worry about it but out here a rubbish truck would be you know or a proper way of removing rubbish all stuff like that so he's the he works for [organisation name] and housing so you know he would be a good too do interview (community member) at the same time...

Interviewer 1: So, with the consent process and the ah I know keep going back and I understand you weren't involved in it but if you were to look at it how would you do that would you do the way so we've done it the right way you think or how would

Well so far I can from Telethon's side you've done um I think it's just you've done it right I I you know like that process with [researcher name] he's been keeping in touch all the time and introducing me to any of your staff um yeah I think if we was I think we need to have more um communication with Local Organisation like Local Organisation come out because usually they just come out do see their clients you know and I don't know what else they do but then you just then they're gone again you know and I it's not their fault either you know but if we get together then do more of that what'd call it networking get together you know even if they can just bring out some sausages and stuff...

Interviewer 2: I'm wondering you as well know Local Organisation was who we picked for this project because they had some links into community but is there you know what it could be someone else or could it be improved if it was someone local or is it more about if even they're external as long as they have good contact with community what's your preference

Interviewer 1: see would you be happy for me to come in and do the consent process or somebody external or doing their consent do you think that like someone outside would have the language and be able to translate what it's all about well enough by having an outside person come in and do it or

Well, if then like now we'd like I'd like to be somebody stand beside that service you know like you've got me (Interviewer 1: so, someone that's local) yeah (Interviewer 1: working alongside like a staff or a staff/staff so your own people are getting trained and is that what you're saying) yep

Interviewer 1: why would that make it better

Because it's that person is here, and you know you don't have to rent anything out or and you've got a base in the area so that you know like all that other services you got [organisation name] you've got they've all got their own offices and but maybe like cause Service Provider is part of it you know. Yeah, we've got to really break down that communication

Interviewer 1: More information sessions…It's like when you launch something I suppose when you make a launch of a project that you'd be really formative upfront to provide the information session up front and the way you get everybody together is probably by food so everyone comes along and listens to what you've got to say see that's kind of like what I'm hearing in what community member is saying um but yeah just when you do the consent who does it better you know I mean it's just refreshing to hear that when they come and do that consent process they have someone working alongside them um that's local from the community to be able to work with them which wasn't the case but she managed to get most of the people to sign up. Be interesting to find out whether the people the mothers particularly knew what they were signing up for

Um yeah that's what makes me wonder too

Interviewer 1: She had a really informative flipchart that she worked from - have we got one (Interviewer 2: yeah, there's one in there)

But you know there's people here if you're not up here with that big picture stuff everyone just lives day to day you know

Interviewer 1: yeah, we talked about that um people just live

Interviewer 2: I was wondering too Researcher you know how we were talking to someone in community yesterday a mother who had signed her kids up for the study and she you know there's so many things you got more important like looking after your kids so how she was forgetting what when Researcher said oh you remember when staff member came out she said oh yeah that's right now I remember how do you know we only hear certain times of the year how do you keep people

Me but nobody uses it but um when yours come out you send a picture of your whole team (Interviewer 2: OK) your photos so before you even get here they'll familiar with that read up all these posters oh this one coming and they'll see you oh you here you know that's how we are Researcher you know that's how Aboriginal people are (Interviewer 2: yeah) they'll see but if they're familiar seeing your face around you know if you put the poster up for a week and when you rock up cause that's the problem half the time (Interviewer 2: who are you) who are you know (Interviewer 1: yeah makes sense yeah) so just a little profile of your little team (Interviewer 2: yeah we that's easy we can do that) (Interviewer 1: yeah that's good) and make it not in um not A4 just on a big A3 (Interviewer 1:/2 yep) so just - we are Telethon in the middle this is our team we're coming again to do this…

Interviewer 2: And so do you think um do you think people need to keep hearing the same message (p14 yeah) over and over again is that to help them learn or as a reminder or

I reckon um we need something like um a TV setup maybe in the shop or in the office where is constantly just showing little ads you know of Aboriginal ads you know just to thing what you what's that smile about (Interviewer 1: you must've been talking to community member name then he said the same thing) I wasn't talking to community member name (laughing) (Interviewer 1: you sure) nah (Interviewer 1: he said the same thing) well that's well that's what I see all the time you know in the shop here (Interviewer 1:/Interviewer 2: mmm) well if they walk in the shop or in the office there'll be something that catch their eye you know (pause) like they have in the clinics... But you know we need to have more of Service Provider involvement in this (Interviewer 1: in what way) because they are on the ground through health promotion because at the moment, I think the clinic is just band-aid. That's where I see it there's not enough of health promotion and I know from lack of um you know them nurses they work hard I’m not knocking them but I'm just saying there's not enough where I see there in the before the nurses used to be out on the community, but I don't know that might be because of the rules of health that they'll not allowed to go back out

Interviewer 1: So, what ya got planned then for the SToP trial what sort of artwork have you got planned you know

Interviewer 1: Story just a story and people can just they'll make a story you know you just give em a book and if you get a group of people together, they'll just sit there

Interviewer 1: So, you think if we had to go and get all the stuff like the canvas and the paint all that you reckon this mob will actually do give us a finish product…the painting will then be owned by the institute or by the community

Well, if you buy it, I don't know a copyright thing

Interviewer 1: Well, it won't be reprinted excepting on the shirts maybe

Oh, look a lot of things like that happen here so it can (Interviewer 1: it can be bought back into the community) yeah that'll be fine

Interviewer 2: Yeah, thank you and you have anything else you think is important you want to tell us or ask us
