# Supplementary material for: Starting the SToP trial: Lessons from a collaborative recruitment approach
Source: PLoS One. 2022 Nov 17;17(11):e0273631. doi: 10.1371/journal.pone.0273631 (PMC9671300; doi:10.1371/journal.pone.0273631)
Supplement: Attachment — Submitted filename: 20210503.PLOSONE (4).docx [file pone.0273631.s004.docx]

Starting the SToP: Lessons from a Collaborative Recruitment Approach

*Tracy McRae^1,3¶^, Roz Walker^1,2¶^, John Jacky^3¶^, Judith M Katzenellenbogen^1&^, Juli Coffin^3&^,

Ray Christophers^6&^, Asha C Bowen^1,3,4,5&^.

Affiliations

^1.^	School of Population and Global Health, University of Western Australia, 35 Stirling Highway, Perth, Western Australia, 6009, Australia

^2.^	School of Indigenous Studies, Poche Centre for Indigenous Health Research, University of Western Australia, 35 Stirling Highway, Perth

^3.^	Wesfarmers Centre for Vaccines and Infectious Diseases, Telethon Kids Institute, 15 Hospital Road, Nedlands, Western Australia, 6009, Australia

^4.^	Perth Children’s Hospital, 15 Hospital Road, Nedlands, Western Australia, 6009, Australia

^5.^	Menzies School of Health Research, Rocklands Drive, Tiwi, Northern Territory 0810, Australia

^6.^	Nirrumbuk Environmental Health and Services Pty Ltd, 34 Blackman Street, Broome, Western Australia 6725, Australia

*Corresponding Author

[tracy.mcrae@telethonkids.org.au](mailto:tracy.mcrae@telethonkids.org.au) (TM)

^¶^ These authors contributed equally to this work

^&^ These authors contributed equally to this work

## Abstract

### Objective:

Recruitment in research can be challenging in Australian Aboriginal contexts. We aimed to evaluate the SToP (See, Treat, Prevent skin infections) trial recruitment approach for Aboriginal families to identify barriers and facilitators and understand the utility of the visual resource used.

### Methods:

This qualitative participatory research used purposive sampling to conduct six semi-structured interviews with staff and five yarning sessions with community members that were audio recorded and transcribed verbatim before thematic analysis.

### Findings:

Recruitment facilitators were existing collaborative partnerships and recruiters known to or living in the community. A prolonged process, under-developed administrative systems and stigma of the research topic emerged as barriers. The pictorial flipchart clearly explained the SToP trial rationale.

### Conclusion:

Partnering with a local Aboriginal organisation, employing Aboriginal researchers, and utilising flip charts for recruitment was seen by some as successful. Strengthening governance with more planning and support for recordkeeping emerged as future success factors.

### Implications for Public Health:

Our findings validate the importance of partnership for this critical phase of a research project. Recruitment strategies should be co-designed with Aboriginal research partners. Further, recruitment rates for the SToP trial provide a firm foundation for building partnerships between organisations and ensuring Aboriginal perspectives determine recruitment methods.

## Introduction

Tackling childhood diseases has the potential to make a substantial contribution to reducing the known disparities in life expectancy between Aboriginal and non-Aboriginal Australians^1^. The burden of skin infections (scabies and skin sores) is highest in newborn babies and children, posing significant public health concerns^2^. Remote living Australian Aboriginal children have the highest reported rate of skin infections globally with almost half the children (45%) suffering from skin sores at any one time^2^. Impetigo (skin sores) is a highly contagious, antibiotic treatable bacterial skin infection following minor trauma, scabies, and insect bites where the skin breaks allow entry of aggressive bacteria^3^. Scabies is a skin infection caused by mites that are transmitted between humans through skin-to-skin contact^4^. Itching and scratching of scabies facilitates the introduction of the bacteria with resultant impetigo^5^.

Environmental and social factors are major determinants of infectious diseases including skin infections, particularly tropical and humid environments, poverty, and limited access to resources^6,7^. Poor housing hardware^7^, limited bedding supplies^6^ and overcrowding^7^ also contribute to the burden of skin infections. Because skin infections are often “normalised” in Aboriginal children^8^, increasing awareness and treatment of skin infections, particularly in populations where impetigo and scabies are widespread, is a priority^8,9^. In the Australian Aboriginal context, this requires a comprehensive transdisciplinary approach encompassing social and behavioural lenses that compliment biomedical and health sciences while privileging the knowledge of community members^10^. There is a particular need to acknowledge the enduring effect of colonialism on health and wellbeing through the loss of culture and identity, land dispossession and racism^10-13^.

This research project is embedded within the See Treat Prevent (SToP) trial, a stepped-wedge cluster randomised trial involving four community clusters in the Kimberley region of Western Australia (WA) aimed at reducing the burden of skin infections in Aboriginal children living remotely^14^. The trial uses a Community Participatory Action (CPAR) approach^15^ focusing on co-design of resources, project governance and data sovereignty to ensure Aboriginal people govern the collection, ownership and application of data about their lands, communities and resources^16^. During the early stages of the SToP trial (2016/2017) and upon recommendation from the Aboriginal partner organisations, recruitment was outsourced to an Aboriginal partner organisation with strong existing relationships with the communities. Also, during this time, it was agreed by SToP trial stakeholders and partners that a visual resource would be used in recruitment to explain about skin infections and the rationale for the SToP trial. Hence, the flip chart was developed and modified with feedback from the research team, Aboriginal stakeholders and SToP trial partners, and clinic staff.

The elements of the SToP trial (Fig 1) include: community consultation/engagement over two years from early 2016, recruitment commencing in 2018, school-based surveillance of skin infections in 2019; treatment of skin infections; and prevention of skin infections through community-driven health promotion and environmental health initiatives. The primary outcome of this trial is a 50% reduction in impetigo in school aged children screened at school^14^. Through their governance, Aboriginal people are co-investigators in all phases, including - and especially relevant to this manuscript – recruiting participants in the SToP trial.

*Figure 1. SToP trial and current research project*

**Skin Surveillance**

**Treatment**

**Prevention**

**Outcome**

**Consultation**

**Recruitment**

**2016 Ongoing**

**September 2018**

**May 2019**

**October 2019 – December 2021**

**2019**

**‘Starting the SToP’**

**Current Project**

**School-based Surveillance**

**2022**

Indigenous people have arguably been the most researched people in the world with little opportunity to provide input to why and how the research is conducted^17^. Consequently, some Indigenous peoples are sceptical about the research process and the methodologies used^18^. In an Australian context, this has frequently resulted in Aboriginal people feeling disempowered^10,11,18^. However, research has reported Aboriginal people can be willing to participate if they perceive the research is beneficial for the community as a collective^19^ Addressing determinants impacting skin infections can only occur through adopting research methodologies that facilitate meaningful partnerships, two-way (reciprocal) learning approaches and involvement of Aboriginal people in all elements of the research process^10,12^. Two-way learning can shift power balances in the broad research team, and strengthen non-Indigenous researchers’ capacity, and knowledge of Aboriginal culture^12^. A CPAR approach enables decolonisation, knowledge sharing and promoting social change^15^, providing an appropriate framework for research involving Australian Aboriginal people^11^.

Recruiting participants into research and clinical trials can be challenging for researchers, particularly when engaging people from culturally diverse backgrounds different to their own^20-25^. Fear of judgement and stigma of the research topic can create challenges for recruiters^23,24,26^. However, building trusting relationships with stakeholders and communities^20,27-30^, employing Aboriginal people^20,27,28^ and using resources during recruitment to explain the research topic and rationale to participants in a meaningful way can help overcome these difficulties^22,28,31^. In Australia, it is an ethical requirement to provide consent and information forms in plain English to potential participants during the recruitment process^32-34^. Culturally appropriate pictorial flip charts can be an effective communication method.^31,35-37^. Displaying information in a pictorial format as opposed to text has been documented as a preferred method for translating health literacy in the Fitzroy Valley in WA^28^.

Despite previous research reporting recruitment barriers and facilitators, there is limited published studies reporting the perspectives of Aboriginal people involved in the recruitment process^32^. Therefore, this research has two objectives:

1. To understand what facilitators and barriers impacted recruitment from the perspectives of the community members, recruiting staff, and researchers.
2. To assess whether the co-designed flipchart was considered a useful tool from the perspective of the community members for informing parents about the nature, prevention, and treatment of skin infections.

## Methods

### Study design and theoretical frameworks:

This qualitative project involved a process evaluation^38^ of the SToP trial recruitment approach. Process evaluations of health interventions investigate how programs or research initiatives are implemented and the critical success factors involved^38^. This study acknowledges the importance of Indigenous standpoint theory^17^ and seeks to bring this standpoint into the analysis through discussions with Indigenous researchers and community members. This study was undertaken by a student researcher (TM) as part of a Master’s dissertation at the University of WA. As a non-Aboriginal raised in New Zealand, TM acknowledges the worldview, conscious values, and privilege she brings to this research and is committed to the constructivism philosophy^39^. This has required adoption of a critical reflexive stance and awareness of the enduring effects of colonialism that continues to impact the lives of Aboriginal people today. Throughout this research, TM received cultural guidance, knowledge and mentoring from Aboriginal co-authors, John Jacky (JJ), Juli Coffin (JC), and Ray Christophers (RC). The three non- Aboriginal co-authors provided mentorship and supervision as follows; qualitative research knowledge Roz Walker (RW); project planning Judy Katzenellenbogen (JK), understanding of skin infections Asha Bowen (AB). It is with their ongoing guidance and support; this research was possible.

Constructivism, a theory of knowledge and learning that proposes multiple perspectives, worldviews, and realities exist^39^, is the philosophical epistemology underpinning this process evaluation. Situating constructivism within an ecological framework helps to understand how individuals’ knowledges and worldviews are constructed through their interactions between physical and social environments existing at the individual/family, service provider and policymaker levels^37^. This research project uses an intrinsic case study^38^ approach to evaluate the SToP trial recruitment process. Intrinsic case studies focus on the richness and complexity of a particular case with less focus on the findings representing other cases^38^. In this instance, the case study encompasses nine remote Aboriginal communities in the Kimberley and the collaborative partnership involved in the SToP recruiting process. An intrinsic case study was appropriate for this research project given the multiple sources of data generated through semi-structured interviews, yarning sessions, and secondary data documentation^38^.

Selection of participants:

All participants in this qualitative research project were recruited using purposive sampling to create a heterogenous sample within and between the two groups of participants, comprising staff and community members^40^. Staff members were recruited by TM, while community members were recruited by JJ. All participants provided written informed consent prior to participating in this research.

Data collection and analysis

Semi-structured interviews with staff from Telethon Kids Institute and operational staff with the Aboriginal partner organisation involved in the recruitment process were conducted by TM to deeply explore their experiences^39^. One-on-one yarning sessions that facilitated informal discussions and sharing of stories and experiences were conducted by JJ with community members from four of the nine communities involved in the SToP trial. Yarning sessions are deemed an appropriate and culturally safe method of gathering data that has a valid place alongside other Western research methods^41^. Semi-structured interview guides were piloted with a small sample of Telethon Kids Institute staff. Yarning session questions were piloted with two Aboriginal community members prior to being used in this research.

All six semi-structured interviews and four of the five yarning sessions were audio recorded using a handheld Olympus audio recorder. Audio files were uploaded into NVivo 12, computer-assisted qualitative data analysis software and transcribed verbatim with any identifying information removed. Project officer observation reports (POORs) completed by SToP trial staff during previous fieldtrips to communities were reviewed. Those POORs providing historical context to the recruitment process were also uploaded into NVivo 12 ensuring all identifying information was removed. An inductive thematic analysis of the data including POORs situated within an ecological framework was conducted. Analytical rigour was obtained using the criteria of credibility, dependability, transferability and confirmability recognised by Guba and Lincoln^42^. Emerging codes and themes were checked using an iterative process that involved discussions with the primary supervisor (RW) and member checking with participants.

## Ethics

This qualitative study to examine recruitment processes was approved by the health ethics review committees at the Child and Adolescent Health Service (Approval number RGS0000000584), the Western Australian Aboriginal Health Ethics Committee (Reference number: 819) and the University of Western Australia (Reference RA/4/20/4123).

## Results

### Participants

A total of six staff and six community members participated in individual interviews and yarning sessions about the consent process for the SToP trial. All staff members were female with varying roles in the SToP recruitment process, from Chief Investigator to recruiting field staff. All staff had undergraduate degrees and four had completed or were currently undergoing post-graduate level education, with one identifying as Aboriginal. Three of the six staff members had previous experience in recruiting participants for research. One male and five female Aboriginal community members participated in yarning sessions.

In order to achieve the objectives of the study, the questions were grouped into four topics: facilitators, barriers, the use of the culturally appropriate flipchart, and strategies for effective and culturally responsively engagement with communities in research.

## Facilitators for SToP trial recruitment

### Collaborative Partnerships

Collaborative partnerships emerged as a critical success factor for recruitment. From the total population of school-aged children across the nine communities, 740 children (~70% of target population) were recruited into the SToP trial study. These statistics were derived from SToP trial records. Informal feedback from recruiting staff indicated families who declined to participate in the SToP trial felt safe in doing so. All staff in this research project confirmed that the leadership, connectedness, and credibility of the partner organisation was essential to achieving high participation rates during recruitment.

*“…I think it [is] essential, I just think we would [not] have gotten the consents without [partner organisation]” – Staff Member P1*

*"[Recruiting staff member] has been effective in being able to really understand who is who, and who to go to, [Recruiting staff member’s] mannerisms as well and the way [the partner organisation staff member] can talk through the consent [process] – Staff Member P2*

*“I highly suspect that the numbers we got have been quite good and we would not have got that amount of participation and that amount of people saying yes had we not had staff members from partner organisation leading this process” – Staff Member P2*

*“I think it [is] definitely a really positive thing that we actually did what our stakeholders wanted in terms of getting an Aboriginal organisation going and doing what they recommended was the best idea” – Staff Member P3*

*“…I would definitely use this process again, I think we can learn from it, and I think we could improve it dramatically…I do think that the wisdom of the partners to ask us to use people known to the communities to go to their homes and ask for permission to be in the study, is a principle that I 110% support” – Staff Member P4*

*“…I think what worked well was the fact that we got a lot [of] consents, in some communities, well over a hundred percent [of predicted numbers], so that worked well” – Staff Member P5*

Community members frequently acknowledged the importance of having an Aboriginal partner organisation leading the recruitment process as they felt the recruiting staff were respectful in their approach which facilitated cultural safety.

*“…They [partner organisation staff] came knocking on the door [and gave] us information about it and asked us if [like the paper document explained] we wanted to do it, be in the trial or not to be either way. But they [were not] pushy or anything, some people [community members] when they see, especially in communities when they see non-Aboriginal people rock up to the door they just want to shut the door and go back inside, they do [not] want to talk…” – Community Member P9*

Community members confirmed the recruiting staff provided information about the SToP trial effectively, using language and visuals that were easy to understand, creating substantial awareness regarding the importance of the SToP trial in their communities.

*“[Yes], we went through that chart [pointing to flipchart], we were all sitting around most of us were mothers and there were other people like [community member name] and [they] were involved in that meeting that recognised things [skin infections]” – Community Member P10*

*“Yeah, they [partner organisation staff] [came] into the school and present[ed] something [for] the kids and some of the parents were there, so they were aware that it was [going to] happen in the community. Some people they just rock up and they want to do all of these things but [provide] no notification or information. [This way of doing it] was really good” – Community Member P9*

Community members also confirmed the value of the collaborative partnership and the employment of Aboriginal people to explain the research topic in Kimberley Kriol, a local language spoken in many communities

*“…It [SToP trial information] was [not] hard for me to understand [recruiting staff member] used words like Kriol…” – Community Member P8*

### Community relationships

Personal relationships, including direct familial relationships between community members and recruiting staff was identified by four staff and two community members as the critical success factor in the recruitment.

*“I think [recruiting staff member] really drove the whole process…and the local knowledge that [recruiting staff member] had and the relationships that [recruiting staff member] had in community was really the only way that we could identify families [not] in the trial, yes, that was really good” – Staff Member P1*

*“…When the [recruiting] person either knew the community really well, then I think we were more successful with those [participation] numbers compared to those where they did not really have that same connection. So, I think, it is two of those things: [having] that connection to the community and also that connection to us that strengthened the recruitment success” – Staff Member P2*

Participants indicated these existing relationships influenced participation rates and accelerated the transfer of information about the SToP trial and consent process on the ground.

*“I think where there was a person leading it [recruitment], who was connected to the community, the consent rates were much higher. I think what [was] reported anecdotally [was that] it was much easier to communicate the trial…” – Staff Member P3*

*“…I think it worked really well [when] the communities where someone knew the person who was asking permission...When there was a relationship in place already, the understanding [of the trial] happened really quickly” – Staff Member P4*

*“… it's good for Aboriginal [people] like [partner organisation staff] to come out and talk because we know some of the [those] workers too, they [are] countrymen you know…” – Community Member P9*

*“…[it works well] at least having a person there [in community] that gets you [researchers] past that door literally to get you in and…it's the first thing so they [community members] get comfortable with [researchers] [community members can say] oh yeah we know that mob you know, [you] just [need] that ice breaker to say this mob [is alright, it is] right talk to them” – Community Member P10*

### Barriers for SToP trial recruitment

Barriers emerging from the data included: staff perceptions that the consent process was protracted; recordkeeping systems to support recruitment were incompletely developed by the research organisation prior to implementation; limited information sharing occurred between partner organisations due to concerns with privacy legislation compliance; and the stigma associated with skin infections and scabies, identified by community members.

### Protracted Process

This was compounded by the many competing demands of daily life in remote communities.

*“…It certainly wasn't just [a] walk up [to] a house once and expect there to be a whole family there who was happy to sign over to the SToP Trial…[recruiting staff] definitely had to do a lot of visits and it was a lot more work. So we've got people who've consented in September [and] October 2018, and the screening [school surveillance] happened eight months later, so [with] that time lapse, people have forgotten what they've consented to and they [community members] didn't necessarily realise that with [recruiting staff], they were actually consenting for Telethon Kids…The community didn't draw that connection, so when we walk[ed] up saying we're doing the SToP trial, people didn't join the dots [between the partnership]…” – Staff Member P3*

*“…I also think that because it took twelve months for the consent to happen, there's so much information and understanding that gets lost over that time, so was there a way that we could have done it faster? Should we have thought about having more people do it more quickly?” – Staff Member P4*

As per POOR reports and the study protocol, the 12-month time allocation to achieve optimal consent was based on an estimated one hour per family discussion using the flip chart and asking parent’s permission to consent their children into the study. Due to a range of circumstances including the wet season (December – March), community mobility associated with cultural obligations (lore and sorry business), and school holidays, multiple visits were required to achieve wide community coverage which contributed to the perceived delays raised by some staff. Additional logistical planning, staffing, travel, and financial costs were required to address these circumstances. Despite this, high participation rates (~70%) were achieved.

*“Communities are in sorry business (mourning) and no travel to communities for non-essential services is permitted or appropriate until further notice. [Recruiting staff] will not be visiting these communities for consent collection until the new year starts” – POORs 13/12/2018*

*"Confirmation from lore boss who advised to keep researchers from entering [community name] and [community name] at least until further notice” – POORs 06/02/2019*

*“[It has been] difficult for the team to get any consents collected over the holidays as most people had left community, so the process of door-to-door wasn’t working.” – POORs 31/01/2019*

*“reported by [recruiting staff] via email, collecting consent has been really slow over the wet season and school holidays as people are away from community…” – POORs 25/01/2019*

*“…[Next time we should] do it [recruitment] really, really targeted [I] probably would've done it a whole lot closer to the actual time of the intervention…to the time of the trial starting, 'cause there's been a huge gap so I probably would've thought a better way to approach [recruitment] is to have a team that can spend a week in one community, like a week prior to the actual visit [intervention] starting or something like that and really intensely do it, rather than having it dragged out by I think one or two people for a whole year” – Staff Member P3*

### At the organisational level, despite substantial planning, staff articulated that the time from initial recruitment to study commencement was prolonged creating flow-on challenges. The POOR reports show it was difficult for the coordinating staff to anticipate the consequence of having an 8-month time period between the start of recruitment and commencement of the actual trial. This relatively protracted recruitment timeframe impacted on the connectivity with, immediate relevance of and reception for the SToP trial when it commenced in May 2019.

### Complex Logistics Relating to Paperwork

Staff identified administrative and record keeping challenges initially experienced in conducting and monitoring of the consent process. Staff described the difficulties of collecting consents and recruiting participants in a remote fieldwork context including misunderstandings in communication and lack of clear record sharing protocols between partners.

*“It's tricky at times because there are not necessarily house numbers or you don't know exactly who signs off on forms and dates of birth are not always known for children, and so some of those items that are usually pretty easy to fill out in research when you're not in these environments on a consent form and that kind of thing were much more difficult. So that affected logistics and made it a bit tricky for those on the ground and for us interpreting the forms that we got back, at times, but I think everyone did a great job at getting as much information as possible…” – Staff Member P2*

*“So we didn't have good systems in place for how we [were] going to get the consent forms, what we [were] going to do with them when we [received] them, how will we [were going] to identify the houses that [recruiting] had already [visited]” – Staff Member P3*

*“I think there was a bit of miscommunication” – Staff Member P5*

Staff members indicated that the unavailability of community house maps and privacy concerns meant that it was difficult for research and recruiting staff to access or share accurate information about children resident in the communities to cross-reference for consent completeness.

*“…so I think that [knowing what houses had been visited] was probably challenging for all of the [partner organisation] staff as well our own staff, we just were [not] sure had people been asked and declined or had they never been asked or had they been visited and weren't home?” – Staff Member P4*

*“…[there was a] misunderstanding [around] recordkeeping, [and if] records were supposed to be kept…partly because [of the] concern around the privacy…many communities don't have a house map per se so that you can document on a map or something visual, [so we were unable to confirm what] houses have already been visited and declined or consented” – Staff Member P4*

### Learning and refining the processes in an iterative manner

The complexities experienced by the recruiting staff were not initially recognised by the Telethon Kids Institute team who assumed their two-day training program would be sufficient to ensure understanding of the partner organisation of the research design and record-keeping requirements. Telethon Kids Institute staff did not always understand the partner organisation’s priorities; however, with ongoing review and reflection by staff throughout the recruitment phase the development of systems to improve record keeping processes improved over time. Problem solving and two-way learning together by staff in both organisations enabled improvements to be implemented to address challenges.

*“…I think that there was the ability to keep on redirecting and reframing and improving the process [that] happened throughout the twelve months. I think it got better and better and probably faster and faster” – Staff Member P4*

### Stigma and Fear of Judgment

The SToP trial has a clear focus on seeing, treating, and preventing skin infections. Initially some community members were concerned with the stigma that can be associated with skin infections and scabies for fear of being judged.

*“The word scabies [is an issue] when describing the trial as people feel it is an insult to their personal hygiene” – POORs 31-01-2019*

*“… there are people who [do not] want to participate as they do [not] have scabies and did [not] want to be labelled as having scabies. [We should, therefore, refocus the trial from a positive perspective and explain] to people that we want to know about everyone’s skin health, including the people who do not have scabies/sores. That will help us to figure out what some people do differently, why some people get these problems and other people do [not]” – POORs 26-02-2019*

The POORs and community members indicated that some families were reluctant to provide consent as they may have been seen by others as having a dirty house or being unclean. In addition, there was a fear of judgement experienced amongst some community members relating to how they look after their children. This highlighted the need for a sensitive approach from researchers, as well as reframing the research to a strengths-based healthy skin focus.

*“[There is] the need for a strengths-based approach to the language we use for the trial. When we describe it as a skin sore/scabies trial, people think it is only for people with skin sores and scabies. If we call it a healthy skin trial, then it applies to everyone” – POORs 26-02-2019*

*“I gave out a few consent forms for [recruiting staff member] and people [were] still reluctant to actually participate in the program thinking that [people] might have been judging them for not looking after their kids or whatever…” – Community Member P10*

*“…Locals think that we [organisations] are scrutinising the way they live, or we are judging them or whatever, so they put up all these defences and barricades…” – Community Member P10*

Flip chart used in SToP trial recruitment

The pictorial flip chart, designed by the SToP trial research team and modified with feedback from SToP trial stakeholders, partners, and clinic staff was used as a visual resource to explain skin infections for recruitment and implementation of the SToP trial. While the flip chart may not have been consistently used during recruitment into the SToP trial, community members affirmed it had been useful for explaining skin infections.

*“[No] not that particular chart but I think they had the paper [participant information sheet]” – Community Member P8*

*“These are good [pointing to flipchart] if you [are] doing one on one stuff because visual is always good for us mob, [it is] really plain and simple, people can relate to that, [they can say], oh, we've seen that sore on our kid but we didn't know what it was” – Community Member P10*

Staff described how the flip chart was developed using a co-design process to determine content and materials.

*“So the flip chart was always in place as a concept from the beginning, but over time, it was morphed and modified using feedback from the clinic, the research team, the partners, the Aboriginal stakeholders, and different people who have informed what that flip chart looks like” – Staff member P4*

Staff also articulated how the flip chart had been effective for explaining to the children how and why skin and throat swabs were going to be taken during school surveillance visits.

*“…So we had a [flipchart] and it’s good for that [explaining skin infections and surveillance to the children], we always had it in the room so there were pictures of people swabbing and taking temperatures, so if the kids kind of looked a bit unsure, we would show them the picture and explain…” – Staff P1*

## Strategies for future recruitment

Staff and community members were asked to reflect on strategies to improve the research recruitment process. Strategies for future research suggested by participants responsible for operational aspects of recruitment included employment and training of local community members in research topics and processes so they feel confident to conduct recruitment and prioritising the joint identification of teams and partnerships through team t-shirts and posters.

### Local Community Members

Employing local community members to conduct recruitment emerged as a key theme perceived by six staff members interviewed as critical to the overall success of recruitment.

*“…Having someone and upskilling people locally on the ground or finding someone who actually lives in that community would probably be really helpful… - Staff Member P2*

*“I think it would have been a much more effective strategy to use community members” – Staff Member P3*

*“So [I] would do it the same, I hope that when we get to do the next study, we will have a much richer web of knowledge and engagement with the communities [so] there are people in place who we can readily employ to do this kind of work…[That will be] good for them [community members] because it [is] employment and capacity-building, and [it is] involved in informing information about their families” – Staff Member P4*

*“I think the best way moving forward would be for community members to have a good broad understanding of research, and then to have a particular training in the project that they are then employed to go and get the consents for. What we need in the future moving forward [is] for community members to feel able to take on this role” – Staff Member P5*

Three out of six community members also reiterated the importance of utilising local people for the process as this would contribute to self-determination and empowerment to facilitate positive and sustained changes to the health and wellbeing of families in their communities.

*“… I reckon working with the community, having people in the community do that, I think… [I would] like to see more and more of our people in the medical industry like having nurses and doctors that are Aboriginal people. We [have to] be proud [have] a bit of self-determination there and be happy about it, see someone else doing something good with their lives” – Community Member P8*

*“If you got someone who lives on community as a navigator going to do [recruitment], it [will] only take a day or really half a day ‘cause they know where everybody is and then you [are] saying we could do [it] as an individual way of approaching so everybody gets touched on but we could also do it on a collective community meeting as well” – Community Member P9*

*“In the community [a local person who can tell community members] [the organisation] is all right, it's fine we can do this, it's only about this [research topic] we [are] not talking about how you mind your kids we [are] just telling you, [to] give you this information”. – Community Member P10*

*“You [cannot] have someone from [this community] go to [Community Name] or [Community Name], you need their own people to do it. So, somebody like for example, [if it is] a health thing [that] would be our local health workers maybe” – Community Member P10*

### Team Branding

The need to promote recognition of the SToP trial and partnership between the organisations emerged as a key issue within these communities and strategy for future projects. The separate roles the partners played – with the local community organisation leading recruitment and Telethon Kids Institute staff implementing the SToP trial, was not readily recognised or understood by community members. Study participants suggested that the recruitment process would have been more effective if branding and identification of the partnership had existed. Both staff and community members suggested that having SToP trial t-shirts and posters to promote the teams and partnerships would have helped community members become familiar with research teams visiting their communities throughout the study.

*“…I think maybe having a mixed team and more shared branding. I think we got the idea right of not having the actual research team doing it [recruitment] ourselves, I think having that little bit of separation [but] I think maybe we were separated a bit too far though” – Staff Member P3*

*“I think what we did [not] really appreciate was any form of branding that might be needed, so we did [not] badge the people with a T-shirt that said we [are] part of the SToP trial and we [are] aligned with Telethon Kids. So, I think that it probably relied on the skills and talents of the individuals who were employed by [partner organisation] much more so than the support that either of the brands [were] able to give. And, [we could] probably learn from that next time… creating a T-shirt that was for the team who was delivering the consent” – Staff Member P4*

*“But when you come out you send a picture of your whole team, your photos, so before you even get here they're [community members] familiar with all these posters, [they will say] oh, this one [person is] coming and they [will] see you… that's how we are, that's how Aboriginal people are. They [will] see but if they're familiar seeing your face around, if you put the poster up for a week and when you rock up ‘cause that's the problem half the time - who are you, you know? So just a little profile of your little team” – Community Member P14*

## Discussion

This project has demonstrated the value of adopting empowering and participatory methodological approaches^10-12,17^ throughout research. The project’s methods encompassed constructivism, Indigenous research methods, and CPAR; situating the interpretation and analysis of findings within an ecological framework. In doing so it has recognised the participants involved in the study are living in communities where historically health research has been conducted without their engagement and often with little benefit to them^10,13,17^. Understanding how broader policies and institutions including research have often adversely impacted Aboriginal people’s wellbeing, was the primary rationale underpinning the research process and analytical framework. Fundamental to the de-colonising approach of this research was the two-way learning relationship^12,41^ between the student researcher TM and Aboriginal researcher and co-author JJ who shared their knowledges. This two-way learning relationship facilitated a culturally secure^43^ environment for yarning sessions where community members were able to openly share their stories with a non-Aboriginal researcher present. The community members’ perspectives and experiences reflected in this research, particularly around stigma and fear of judgement, highlight the ongoing legacy of colonisation. While several studies confirm the benefits of working in collaborative partnerships with well-respected local Aboriginal organisations underpinned by strong Aboriginal governance to improve recruitment and research outcomes^20,21,25,27-30^, limited information is reported on the experiences of Aboriginal people involved in the recruitment process. This research provides insight from the perspectives of Aboriginal community members into how recruitment should be conducted in their communities.

As a result of the insights from this qualitative study, the importance of stronger research partnerships in which Aboriginal organisations have shared leadership of the project and process, including recruitment is revealed. We have learned there needs to be a commitment to better governance, partnership building, greater commitment to co-design and equal ownership of continuous improvement through-out the partnership. Formalised partnership pathways should be in place well before recruitment is scheduled so that the local Aboriginal service partner has ‘shared lead’ for development of the recruitment process as relevant to the specific location and specific study or project.

Interviews and yarning circles with staff and community members involved in the SToP trial recruitment process highlight several facilitators and barriers to obtaining participant consent. The key facilitators included the outsourcing of recruitment to an Aboriginal partner organisation with strong cultural ties to the communities involved, on the advice of the Aboriginal leaders in the SToP trial partnership. This approach was based on extensive consultation with key stakeholders and the partner organisation facilitating the recruitment to ensure a culturally appropriate, community-led recruitment process and is a key strength and recurrent theme consistently identified by the evaluation.

The culturally appropriate co-designed flip chart which incorporated both the study logo and logos of all partner organisations was well-received by community members and helped staff to explain skin infections during school surveillance visits. In addition, identification of teams and partnership through ‘branding’ was also recommended as a strategy. Whilst the SToP trial and partner organisation logos were present on all study paperwork, the staff believed a more visible SToP trial T-shirt would have provided greater recognition of the partnership and the trial.

A strong theme emerging from the data as a critical success factor was having the Aboriginal partner organisation lead the SToP trial recruitment process. Staff attributed the high recruitment rates to the collaborative partnership where the Aboriginal recruiting staff had strong connections with the communities in SToP. This enables short-cuts to the process by relying on existing trust-based relationships to secure participation. Community members participating in yarning circles also identified the value of this approach, particularly having Aboriginal people explaining the research to them in a meaningful, easily understandable way. All community members confirmed that recruiting staff communicated the SToP trial effectively creating substantial awareness of the research being conducted in their communities.

Rae and colleagues^20^ report similar findings in their ArtsHealth project conducted in Queensland where a collaborative partnership forged between researchers and the local Aboriginal Medical Service (AMS) facilitated recruitment and the success of the project. Fitzpatrick and colleagues^28^ reinforce these findings from their Picture Talk project conducted in Fitzroy Crossing, highlighting the importance of well-respected and trusted organisations supporting research. Similarly, the SToP trial reports the effectiveness of using local language and recruiting staff with personal connections and shared cultural understandings and experiences to the participants.

Consistent with the literature, recruiting staff with personal connections to communities enhanced research recruitment rates^23,27-29^. Staff confirmed that whenever recruiting staff were from, had family relationships or were connected in other ways to a community, participation rates appeared higher. This suggests that recruitment may meet less resistance in communities where prior personal relationships between recruiting staff and community members existed. Similarly, McDougall and colleagues^23^ report initial low participation rates within the African American cohort improved through the connections of a well-known community liaison officer who provided reassurance about the research and assisted with recruitment. The findings from a South Australian study of chronic disease prevalence by Marin and colleagues^27^ also support this, highlighting that connection to community and local community knowledge provided a sound basis for interviewers to engage with participants, who in most cases, were comfortable speaking with someone from their own community. These studies affirm the value of training and employing local community members throughout the research process including recruitment. Investment in relationship building before designing and commencing research to ensure it is a priority for local participants and there are well-respected and trusted community members who receive training and support to deliver the research is a model that should be adopted more fully. This will assist with enhancing community self-determination and empowerment and contribute to decolonising western research approaches.

Culturally appropriate pictorial flip charts to translate health literacy in a meaningful way to Aboriginal people have been actioned in several different projects and proven to be an effective communication method, particularly in the NT^31,35-37^. In the context of skin health, pictorial flip charts have been used in the NT to clinically diagnose skin infections^37^. Similarly, co-developed flip charts have used local language and cultural practices as analogies to explain skin infections to participants during a mass drug administration (MDA) project^31^. These findings are consistent with another study in the NT conducted by Russell and colleagues^36^, where Aboriginal participants preferred research information to be presented in flip charts with visuals. In WA, Fitzpatrick and colleagues^28^ report similar findings from their study conducted in the Fitzroy Valley where focus group participants preferred information expressed in pictures rather than words. While the data from this research did not reveal whether the flip chart was consistently used in recruitment or whether it influenced participation into the trial, positive feedback from participants reiterated the visual resource cut through concepts and language barriers to explain about skin infections and the SToP trial.

Conducting recruitment in a remote context can be a complex and protracted process for external researchers^21^. While researchers on another Kimberley study substantially under-estimated the length of time required (30 months) for their recruitment phase^21^, the SToP trial team were aware that it would be a lengthy process. Whilst this is the rhythm of life in the remote Kimberley, much of the protracted process was predictable. With better planning and a more targeted and coordinated approach, recruitment may have been more easily achieved and completed closer to the actual study commencement. Initial consultations with community leaders involved with the AMS and local councils were conducted by SToP trial staff prior to submission of ethics applications. As identified by staff, there is a need for a more targeted and coordinated approach to conduct recruitment closer to the implementation of the intervention for continuity and relationship building. Recognising the possibility of substantial delays when conducting research with remote Aboriginal communities, a well-constructed engagement strategy to keep the project relevant and contextual whilst navigating research ethics, governance and recruitment is recommended. This strategy was effective for McDougall and colleagues^23^ who recruited participants close to their study implementation which improved recruitment rates as participants’ interest in the project remained high.

The limited administrative organisation, unclear documentation and maintenance of recordkeeping systems during the initial recruitment phase was a strong theme emerging from the data. Unavailability of community street maps and privacy concerns made it difficult for the Aboriginal researchers to accurately monitor which houses had been visited and whether families had consented. Initially, the lack of a methodical approach to paperwork logistics created some confusion and miscommunication between the partners. However, as part of the CPAR approach regular project implementation meetings were conducted where staff discussed recruitment issues and how to address them. Staff reflected on how houses could be more effectively tracked through a more systematic recording of data while still respecting privacy. This resulted in collectively developing a more systematic approach to paperwork logistics which improved the recruitment process over time. These challenges provide insight into the complexities and realities of conducting research in a remote context, identifying the importance of transferring data capture skills from research staff to partner organisations and of collaborative partnerships resolving issues together. While valuable lessons were learnt for the SToP trial, highlighting these challenges and developing strategies to address them significantly adds to the literature for future research projects conducted in remote Aboriginal communities.

Fear of judgment and stigma pertaining to health topics is not a new concept in research^23,24,26^ but an important one for researchers to consider. Addressing the issue of stigma in research involves taking a sensitive approach embedded within a strength-based framework^23^. In Australia, the enduring effects of colonialism continually impacting Aboriginal individuals, families and communities^13^, underly the stigma and fear of judgment surrounding skin infections and scabies in children that emerged as a concern. This reiterates the need for researchers and health professionals to reassure individuals that the study focus is about healthy skin. Acknowledgment of the enduring effects of colonialism requires broaching research topics with sensitivity and non-judgment^23,24^. McDougall and colleagues^23^ highlight stigma as an issue in their recruitment of older participants with dementia. They reported using positive language in media advertisements disseminated with a strength-based approach helped reduce stigma and fear of judgment amongst their study participants. Likewise, Jones and Jablonski^24^ reiterate the need to understand and incorporate the language and terminologies used in communities for specific health issues in a positive and non-stigmatising manner. The findings from the SToP trial support these recommendations where recruiting staff articulated the information to community members using a positive strength-based approach focusing on healthy skin for everyone.

Participants in this research emphasised a future strategy of employing and training local community members on research projects to build trust and relationships between researchers and community members. The strong existing relationships between recruiting staff and a well-respected community member who could navigate their visit and direct them to families in the community, resulted in high recruitment rates. Employing well-respected community members who can share their knowledge and provide cultural guidance will be an essential element of Telethon Kids Institute research projects in the future^28,30^.

The need to promote the partnership through a recognised brand within the community and between the organisations emerged as a key issue and strategy for future projects. The separate roles the partners played, with the local community organisation leading recruitment and Telethon Kids staff implementing the SToP trial, created a disconnect for the community. A suggested strategy from both staff and community members, was the need to identify teams and partnerships through t-shirts and posters to overcome the barriers experienced. This would help community members become familiar with research teams visiting their communities as was reported by Rae and colleagues^20^ on the ArtsHealth project community members. Consistency and familiarity are important aspects of building trust and relationships between researchers and communities as well as maintaining visibility of the SToP trial over a prolonged period.

## Implications for Public Health

Prior research investigating barriers and facilitators for recruitment have reported the perspectives of researchers and staff, with few Australian publications reporting the perspectives of Aboriginal people involved in research recruitment^34^. We contextualise staff reports on recruitment with the richness and privileging of Aboriginal community voices in the SToP trial recruitment process. Further, this project validates the cultural appropriateness of using pictorial flip charts to explain research and translate Western health concepts to Aboriginal people^31,35-37^. The collaborative recruitment approach used in the SToP trial is novel in a remote clinical trial context focusing on skin health. While the SToP trial recruitment was a protracted process that created administrative challenges and raised concern of stigma around scabies and skin infections, the findings support the effectiveness of having a well-respected local Aboriginal organisation lead recruitment and employing local community members as navigators^28,29^. The high rates of recruitment for the SToP trial confirm the need for Aboriginal people to be involved in all phases of the research process. The community members in this research spoke positively about the SToP trial recruitment process providing insightful observations and strategies for conducting recruitment in the future. The findings reported here provide strategies to help inform best practice in future research engaging Aboriginal people in remote and clinical trial contexts.

## Strengths and Limitations

This research project was designed by an early non-Indigenous career researcher with guidance and support from supervisors and Kulunga Aboriginal Research Development Unit team staff in Perth and Broome. Having a local Aboriginal mentor to provide cultural guidance throughout the process was a key strength. While only a small sample participated in the study, the rich narratives resulting from the semi-structured interviews and yarning sessions confirmed by the POORs align with the case study design. This design underpinned by Constructivism and Indigenous research methods embedded within an ecological framework has privileged Aboriginal peoples’ worldviews, to ensure they have a genuine voice in this research, and to provide insightful strategies for future research for themselves and other Aboriginal communities.

## Conclusions and recommendations for future research

This qualitative study reveals the importance of stronger research partnerships in which Aboriginal organisations have shared leadership. Formalised partnership pathways should be in place well before recruitment is scheduled so that the local Aboriginal service partner has ‘shared lead’ for development of the recruitment process as relevant to the specific location and specific study or project.

Recruitment conducted for research in remote Aboriginal communities is complex, but can be successfully undertaken with good preparation, protocols and community engagement. The recruitment process led by Aboriginal people was based on SToP trial key partners’ advice and resulted in a highly effective and culturally responsive approach to recruitment in Aboriginal communities. The findings from this research support CPAR principles of employing local community members as Community Navigators to conduct recruitment. To maximise research participation in remote communities, future research projects should apply a methodical approach to consent collection where recordkeeping systems are rigorous but flexible to allow for the complexities of working remotely. Hard copy paper documents were used during the SToP trial recruitment process, however, mechanisms such as iPads for capturing data and streamlining processes may facilitate better awareness of how recruitment is progressing. Budgeting for the cost and time of recruitment in partnership and using branding such as t-shirts can help maintain awareness prior to the commencement of the study. Planning for engagement during this phase is critical to ensure ongoing visibility and knowledge of the research project. Establishing strong and genuine partnerships outlining clear protocols including confidentiality, data sharing and role expectations and responsibilities through formal partner agreements or Memorandums of Understanding can help mitigate logistical challenges of conducting research in a remote context. Formalising the recruitment and consent process in conjunction with Aboriginal partners throughout all phases from initial co-design of the research to co-authorship is strongly recommended. Further, acknowledging and disseminating the findings and recommendations from this research and maintaining collaborative relationships between partners will inform the implementation phase of the SToP trial.

## Acknowledgements

The authors acknowledge all SToP trial communities, stakeholders, partners and RACP. The authors thank all the participants for sharing their experiences. Funding was received from the National Health and Medical Research Council [NHMRC] (project grant 1128950), Health Outcomes in the Tropical NORTH [HOT NORTH 113932] Indigenous Capacity Building Grant), and WA Health Department and Healthway grants contributed to this research. ACB receives a NHMRC investigator Award (1175509).

# References

1. Australian Institute of Health and Welfare Australia’s health series no. 16. Australia’s health 2018. Canberra 2018 [cited 2019 October 27]. Report No.: AUS 221. Available from: https://www.aihw.gov.au/reports/australias-health/australias-health-2018/contents/table-of-contents

2. Bowen AC, Mahe A, Hay RJ, Andrews RM, Steer AC, Tong SYC, et al. The Global Epidemology of Impetigo: A Systematic Review of the Population Prevalence of Impetigo and Pyoderma PLoS One [Internet]. 2015 [cited 2019 February 21];10(8):1-15.

3. Mahe A, & Hay RJ. Epidemiology and management of common skin diseases in children in developing countries. Geneva: Organisation WH; 2005.

4. Hay RJ, Steer AC, Engelman D, & Walton S. Scabies in the developing world its prevalence, complications, and management. Clinical Microbiology and Infection 2012 [cited [cited 2019 Feb 3]];18(4):313-323.

5. Bowen AC, Tong SYC, Andrews RM, O'Meara IM, McDonald MI, Chatfield MD, et al. Short-course oral co-trimoxazole versus intramuscular benzathine benzylpenicillin for impetigo in a highly endemic region: an open-label, randomised, controlled, non-inferiority trial The Lancet [Internet]. 2014 [cited 2019 January 31];384(9960):2132-2140. Available from: ClinicalKey

6. Thomas S, Crooks K, Taylor K, Massey PD, Williams R, & Pearce G. Reducing recurrence of bacterial skin infections in Aboriginal children in rural communities: new ways of thinking, new ways of working. Australian Journal of Primary Health [Internet]. 2017 [cited 2019 January 31]. Available from: CSIRO Publishing

7. Bailie R.S, Stevens M, McDonald EL. The impact of housing improvement and socio-environmental factors on common childhood illnesses: a cohort study in Indigenous Australian communities. 2012 [cited 2019 June 18]. Available from: https://jech.bmj.com/content/66/9/821

8. Yeoh DK, Anderson A, Cleland G, & Bowen AC. Are scabies and impetigo "normalised"? A cross-sectional comparative study of hospitalised children in northern Australia assessing clinical recognition and treatment of skin infections. PloS Neglected Tropcial Disease [Internet]. 2017 [cited 2019 February 2 ];11(7):e0005726. . Available from: PubMed Central

9. Yeoh DK, Bowen AC, & Carapetis JR. Impetigo and scabies e Disease burden and modern treatment strategies. Journal of Infection 2016 [cited [cited 2019 Jan 23]](72):561-567.

10. Brown AD, Morrissey MJ, Sherwood JM. Uncovering the Determinants of Cardiovascular Disease among Indigenous People. Ethnicity & Health. 2006 [cited 2019 November 01];11(2):191-210. Available from: https://www-tandfonline-com.ezproxy.library.uwa.edu.au/doi/full/10.1080/13557850500485485

11. Wright M, & O'Connell M. Negotiating the right path: Working together to effect change in healthcare service provision to Aboriginal peoples. Action Learning Action Research Association Journal [Internet]. 2015 [cited 2019 June 25];21(1):108-123. Available from: https://espace.curtin.edu.au/handle/20.500.11937/11610

12. D'Antoine H, Abbott P, Sherwood J, Wright M, Bond C, Dowling C, et al. A collaborative yarn on qualitative health research with Aboriginal communities. Australian Indigenous Health Bulletin [Internet]. 2019 [cited 2019 April 12];19(2):1-5. Available from: http://healthbulletin.org.au/articles/a-collaborative-yarn-on-qualitative-health-research-with-aboriginal-communities/

13. Johnstone MJ. Research ethics, reconciliation, and strengthening the research relationship in Indigenous health domains: An Australian perspective. International Journal of Intercultural Relations. 2007 [cited 2019 July 05];31(3):391-406. Available from: ScienceDirect

14. Mullane MJ, Barnett TC, Cannon JW, Carapetis JR, Christophers R, Coffin J, et al. SToP (See, Treat, Prevent) skin sores and scabies trial: study protocol for cluster randomised, stepped-wedge trial for skin disease control in remote Western Australia. BMJ Open. 2019 [cited 2019 November 01];9(e0303635).

15. Wallenstein N, Duran B. Using Community-Based Participatory Research to Address Health Disparities. Health Promotion Practice. 2006 [cited 2019 December 13];7(3):312-323. Available from: SAGE

16. Australian Institute of Aboriginal and Torres Strait Islander. Delivering Indigenous Data Sovereignty. 2019 [2020 Feb 25 ]. Available from: https://aiatsis.gov.au/publications/presentations/delivering-indigenous-data-sovereignty

17. Rigney L. Internationalization of an Indigenous Anticolonial Cultural Critique of Research Methodologies: A Guide to Indigenist Research Methodology and Its Principles. Wicazo Sa Review. 1999 [cited 2019 October 24];14(2):109-121. Available from: https://www-jstor-org.ezproxy.library.uwa.edu.au/stable/1409555?sid=primo&origin=crossref&seq=1#metadata_info_tab_contents

18. Hunt J. Engaging with Indigenous Australia - exploring the conditions for effective relationships with Aboriginal and Torres Strait Islander communities. Canberra & Melbourne: Australian Institute of Family Studies; 2013 [cited 2018 August 21]. Available from: https://www.aihw.gov.au/reports/indigenous-australians/engaging-with-indigenous-australia-exploring-the/contents/table-of-contents

19. Guillemin M, Gillam L, Barnard E, Stewart P, Walker H, Rosenthal D. “We’re checking them out”: Indigenous and non-Indigenous research participants’ accounts of deciding to be involved in research. International Journal for Equity in Health. 2016 2016/01/16 [cited 2020 May 14];15(1):8.

20. Rae K, Weatherall L, Hollebone K, Apen K, McLean M, Blackwell C, et al. Developing research in partnership with Aboriginal communities - strategies for improving recruitment and retention. The International Electronic Journal of Rural and Remote Health Research, Education, Practice and Policy [Internet]. 2013 [cited 2019 February 02]:1-8. Available from: https://www.rrh.org.au/journal/article/2255

21. Marley J, Kitaura T, Atkinson D, Metcalf S, Maguire G, Gray D. Clinical trials in a remote Aboriginal setting: lessons from the BOABS smoking cessation study. BMC Public Health [Internet]. 2014 [cited 2019 February 02];14(579):1-9. Available from: ProQuest Central

22. Hughson J, Woodward-Kron R, Parker A, Hajek J, Bresin A, Knoch U, et al. A review of approaches to improve participation of culturally and linguistically diverse populations in clincal trials. BioMed Central [Internet]. 2016 [cited 2019 March 03];17(263). Available from: PubMed Central

23. McDougall GJ, Simpson G, Friend ML. Strategies for research recruitment and retention of older adults of racial and ethnic minorities. Journal of Gerontological Nursing. 2015;41(5):14-23.

24. Jones C, Jablonski R. "I don't want to be a guinea pig": recruiting older African Americans. Journal of gerontological nursing. 2014 [cited 2019 October 14];40(3):3. Available from: ProQuest Central

25. Carroll JK, Yancey AK, Spring B, Figueroa-Moseley C, Mohr DC, Mustian KM, et al. What are successful recruitment and retention strategies for underserved populations? Examining physical activity interventions in primary care and community settings. Translational Behavoural Medicine. 2011;1(2):234-251.

26. Topp J, Andrees V, Weinberger NA, Schäfer I, Sommer R, Mrowietz U, et al. Strategies to reduce stigma related to visible chronic skin diseases: a systematic review. Journal of the European Academy of Dermatology and Venereology. 2019;33(11):2029-2038.

27. Marin T, Taylor AW, Grande ED, Avery J, Tucker G, Morey K. Culturally appropriate methodology in obtaining a representative sample of South Australian Aboriginal adults for a cross-sectional population health study: challenges and resolutions. BioMed Central. 2015 [cited 2019 September 13];8(200):1-10. Available from: PubMed Central

28. Fitzpatrick EFM, Macdonald G, Martiniuk ALC, Oscar J, D’Antoine H, Carter M, et al. The picture talk project: Aboriginal community input on consent for research. BMC Medical Ethics. 2019 [cited 2019 September 13];20(12):1-15. Available from: PubMed Central

29. Dudgeon P, Scrine C, Cox A, & Walker R. Facilitating Empowerment and Self-Determination Through Participatory Action Research: Findings From the National Empowerment Project. International Journal of Qualitative Methods [Internet]. 2017 [cited 2019 Jan 29];16:1-11. Available from: Sage Journals

30. Haynes E, Marawilic M, Makungun Marikac B, Mitchell A, Phillips J, Bessarab D, et al. Community-based participatory action research on rheumatic heart disease in an Australian Aboriginal homeland: Evaluation of the ‘On track watch’ project. Evaluation and Program Planning. 2019 [cited 2019 December 14 ];74:38-53. Available from: Science Direct

31. Shield JM, Kearns TM, Garngulkpuy J, Walpulay L, Gundjirryirr R, Bundhala L, et al. Cross-Cultural, Aboriginal Language, Discovery Education for Health Literacy and Informed Consent in a Remote Aboriginal Community in the Northern Territory, Australia. Tropical Medicine and Infectious Disease [Internet]. 2018 [cited 2019 April 19];3(15):1-11. Available from: https://www.mdpi.com/2414-6366/3/1/15

32. McGrath P, & Phillips E. Western Notions of Informed Consent and Indigenous Cultures: Australian Findings at the Interface. Journal of Bioethical Inquiry. 2008 [cited 2019 November 01];5(1):21-31. Available from: SpringerLink

33. Australian Goverment. In: Australian Code for the Responsible Conduct of Research. 2007. Canberra: Australian Goverment.

34. Fitzpatrick E, Martiniuk A, D’Antoine H, Oscar J, Carter M, Elliott E. Seeking Consent for Research with Indigenous Communities: a systematic review. BioMed Central Medical Ethics. 2016 [cited 2019 October 10];17(65):1-18. Available from: Pub Med Central

35. McKay CC, Chang AB, Versteegh LA, GB. M. Culturally appropriate flipcharts improve the knowledge of common respiratory conditions among Northern Territory Indigenous families. Health Promotion Journal of Australia. 2015 [cited 2019 October 29];26(2):150-153. Available from: ProQuest Central

36. Russell FM, Carapetis JR, Liddle H, Edwards T, Ruff T, Devitt J. A Pilot Study of the Quality of Informed Consent Materials for Aboriginal Participants in Clinical Trials. Journal of Medical Ethics. 2005 [cited 2019 October 14];31(8):490-494. Available from: PubMed Central

37. Andrews RM, Kearns T, Connors C, Parker C, Carville K, Currie BJ, et al. A regional initiative to reduce skin infections amongst Aboriginal children living in remote communities of the Northern Territory, Australia PloS Neglected Tropcial Disease 2009 [cited 2019 January 3];3(11):e554. Available from: Directory of Open Access Journals

38. Baranowski T, & Stables G. Process Evaluations of the 5-a-Day Projects. Health Education and Behavior [Internet]. 2000 [cited 2019 June 21];27(2):157-166. Available from: https://journals.sagepub.com/doi/abs/10.1177/109019810002700202

39. O'Donnell A. Constructivism. In: APA educational psychology handbook, Vol 1: Theories, constructs, and critical issues [Internet]; 2012 [cited 2019 June 19]. Available from: Ovid Books

40. Gray D. Doing Research in the Real World. 2nd ed. London: SAGE 2009.

41. Bessarab D, & Ng’andu B. Yarning About Yarning as a Legitimate Method in Indigenous Research. International Journal of Critical Indigenous Studies [Internet]. 2010 [cited 2016 November 04];3(1):37-50. Available from: http://www.isrn.qut.edu.au/pdf/ijcis/v3n1_2010/Final_Bessarab_Bridget_IJCIS.pdf

42. Guba EG, & Lincoln YS. Competing paradigms in qualitative research. . In: In N.K. Denzin & Y.S. Lincoln (Eds), Handbook of qualitative research. Thousand Oaks CA: Sage; 1994.

43. Coffin J. Rising to the Challenge in Aboriginal Health by Creating Cultural Security. Aboriginal and Islander Health Worker Journal. 2007 [cited 2020 May 28];31(3):22-24. Available from: https://search-informit-com-au.ezproxy.library.uwa.edu.au/fullText;dn=955665869609324;res=IELHEA
